# Alleviating summer heat stress in cowpea-baby corn intercropping with stress-reducing chemicals and fertility variations

**DOI:** 10.1038/s41598-024-52862-2

**Published:** 2024-02-06

**Authors:** Anju Bijarnia, J. P. Tetarwal, Anil Kumar Gupta, Arjun Lal Bijarnia, Rajendra Kumar Yadav, Baldev Ram, Roshan Kumawat, Monika Choudhary, Rajesh Kumar, Deepak Singh

**Affiliations:** 1 Department of Agronomy, Agriculture University, Kota, Rajasthan India; 2 Department of Plant-Physiology, Agriculture University, Kota, Rajasthan India; 3 Department of Agrostology, Agriculture University, Jodhpur, Rajasthan India; 4 Department of Soil Science, Agriculture University, Kota, Rajasthan India; 5 Department of Agronomy, Maharana Pratap University of Agriculture & Technology, Udaipur, Rajasthan India; 6 Division of Sample Surveys, ICAR-Indian Agricultural Statistics Research Institute, New Delhi, India

**Keywords:** Physiology, Plant sciences

## Abstract

Over the past century, the average surface temperature and recurrent heatwaves have been steadily rising, especially during the summer season, which is affecting the yield potential of most food crops. Hence, diversification in cropping systems with suitable fertilizer management is an urgent need to ensure high yield potential during the summer season. Since intercropping has emerged as an important strategy to increase food production, the present study comprises five intercropping systems in the main plot (sole cowpea, sole baby corn, cowpea + baby corn in 2:1, 3:1, and 4:1 row ratio), three levels of fertilizer viz*.* 100 (N_20_ P_40_), 125 (N_25_ P_50_), and 150% (N_30_ P_60_) recommended dose of fertilizer (RDF) in the subplot, along with two stress-mitigating chemicals (0.5% CaCl_2_ and 1% KNO_3_) in the sub-sub plots. A split-split plot system with four replications was established to carry out the field experiment. The effect of intercropping, fertilizer levels, and stress-mitigating chemicals on crop growth rate (CGR), relative growth rate (RGR), plant temperature, relative water content (RWC) and chlorophyll content of cowpea and baby corn, as well as cowpea equivalent yield (CEY), was investigated during the summer seasons of 2019 and 2020. The experiment was conducted at Agriculture University, Kota (Rajasthan), India. Results showed that CGR, RGR, RWC and chlorophyll content of both crops and CEY were maximum under intercropping of cowpea and baby corn in a 2:1 row ratio compared to other intercropping systems. However, the plant temperature of both crops was significantly lower under this system. CEY, CGR, RGR, and chlorophyll content were considerably greater in the subplots with a fertilizer application of 150% RDF compared to lower levels of fertilizer (100 and 125% RDF). Our findings further show that foliar application of CaCl_2_ 0.5% at the flowering and pod-developing stages of cowpea dramatically boosted CEY, CGR, RGR, RWC, and chlorophyll content of both crops and lowered the plant temperature.

## Introduction

The impact of stress at critical growth stages is a significant hindrance for plant growth and development in various climatic conditions^1^. Temperature stress during crop growth plays a crucial role in determining the crop yield^2^. The climate in south-eastern Rajasthan, India, is characterized by hot summers with frequent rainfall events^3^. Stress conditions during the summer season can lead to modifications in plant growth, morphology and root physiology, affecting ion and water uptake^4^. In north-western India, higher temperatures pose several challenges, including reduced growth rates, decreased yields, and detrimental effects on various physiological parameters, making it particularly challenging to cultivate crops during the summer season due to the elevated temperature stress, which results in higher transpiration losses of water ^5^.

High temperatures and extended dry spells during the reproductive phase of crops in the summer season can lead to excessive floral abscission, resulting in poor pod setting, anther dehiscence, and male sterility^6^. Further, the high night temperatures in north-western India during the reproductive phase of cowpea, consistently above 20 °C, may have detrimental effects on flowering and seed setting^3^. To mitigate the adverse effects of heat, thermo-tolerance can be induced through the exogenous application of chemicals and osmo-protectants. Foliar spray of stress-mitigating chemicals can alter the physiological and biochemical processes of plants, aiding in managing yield reduction in field crops^7^. Osmo-protectants like calcium chloride (CaCl_2_) and potassium nitrate (KNO_3_) promote photosynthesis, hyperplasia, and hypertrophy, positively influencing crop growth by affecting water uptake, root growth, turgour maintenance, and the activation of numerous enzymes under environmental stress conditions^7,8^.

In addition to foliar applications of chemicals, employing cropping techniques that can effectively manage high temperatures during crop growth stages is essential for achieving robust crop growth and high yields^9^. Intercropping is a technique where different crops are grown simultaneously during the same season on the same piece of land^10^. Cereal-legume intercropping plays a vital role in subsistence food production, particularly in situations with limited water resources^11^. It can alter the abiotic and biotic characteristics of agro-ecosystems by incorporating different types of crops, such as maize and cowpea^12^^,^^13^. Numerous studies have shown that an intercropping can enhance the efficiency of capturing and utilizing solar energy, resulting in higher yields compared to sole cropping^14^. The efficiency of legumes in fixing atmospheric nitrogen through symbiosis with rhizobia makes them particularly significant in low-input agriculture^15^. In this study, we focus on cowpea (*Vigna unguiculata L*.) and baby corn (*Zea mays L*.) as they belong to different plant families, with baby corn having tall canopies and cowpea having short canopies. The combination of a legume crop with a non-legume offers yield advantages over sole cropping owing to the presence of distinct canopy architectures^16^.

To achieve maximum productivity in intercropping, the careful adjustment of nitrogen and phosphorus application rate is essential^17^. Hence, these fertilizers play a crucial role in improving grain production, NP ratio, and water usage efficiency^18,19^. Nitrogen is a fundamental input in farming systems, enhancing grain quality and overall crop production^20–23^ and phosphorus is essential for plant structural compounds and acts as a catalyst in key biochemical activities in plants^24,25^. Therefore, their simultaneous application is necessary for maximizing crop production^26–28^. Current recommendations for NP fertilizer application are primarily based on cowpea sole cropping and recommended NP doses of cowpea for regions of Rajasthan and Haryana are 20 kg N + 40 kg P^29^. However, there is a lack of adequate research on N and P fertilizer management in cowpea + baby corn intercropping system.

To assess the impact of various treatments on crop growth and productivity, a comparative study was conducted. This study examined the potential of cowpea when grown as a sole crop, baby corn as a sole crop and three intercropping row ratios of cowpea and baby corn in the field. The assessment included key parameters such as CGR, RGR, chlorophyll content, RWC, plant temperature and CEY. These parameters serve as essential indices for evaluating plant productivity and environmental efficiency. Besides, RWC and plant temperature were considered as crucial indicators of water levels under stress conditions. The primary objective of this experiment was to analyze the effects of intercropping, fertilizer levels and comparing of two stress-mitigating chemicals with each other with no control treatment on growth indices, physiological parameters and CEY in cowpea and baby crop intercropping practices during summer season.

## Materials and methods

### Site description

A field study was carried out during the summer seasons of 2019 and 2020 at the College of Agriculture, Ummedganj, Agriculture University, Kota. The location is situated at an elevation of 271 m above mean sea level, positioned at latitude 25° 13′ N and longitude 75° 28′ E. This area falls within the Central Plateau and Hills zones of India (Zone VIII) and the Humid South Eastern Plain zone (Zone V) of Rajasthan^30^.

### Weather

**Figure 1:**
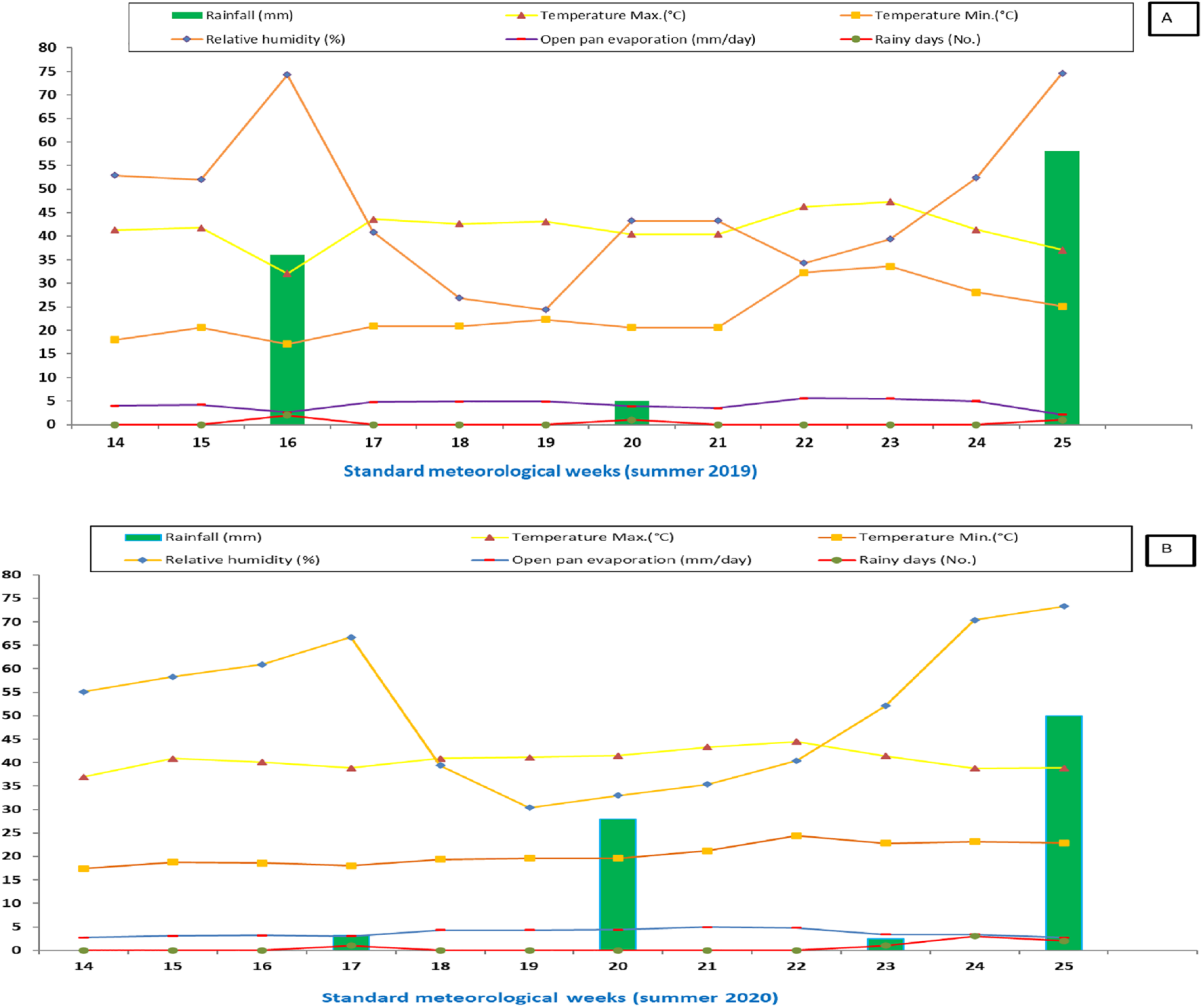
Meteorological data of Kota, India for the cowpea and baby corn growing season (*summer* season) corresponding to the year (**A**) 2019 and (**B**) 2020.

Weather parameters, including temperature, relative humidity, rainfall, evaporation, and rainy days at the experimental site, are depicted in Fig. 1. The data source for this information is the Class ‘B’ Meteorological observatory at the Agricultural Research Station, Agriculture University, Kota, Rajasthan, India.

### Soil properties

The soil in the field experiment had a clay loam texture, consisting of 22.62% sand, 37.67% silt and 34.72% clay. It was relatively deep and had good drainage properties, with an average bulk density of 1.26 Mg/m^3^. The soil in the experimental area had moderate levels of organic carbon (0.52%), available nitrogen (313 kg/ha) and phosphorus (23.05 kg/ha). Its pH was slightly alkaline at 7.6 and it contained a high amount of potassium at 394 kg/ha.

### Experimental detail

**Figure 2:**
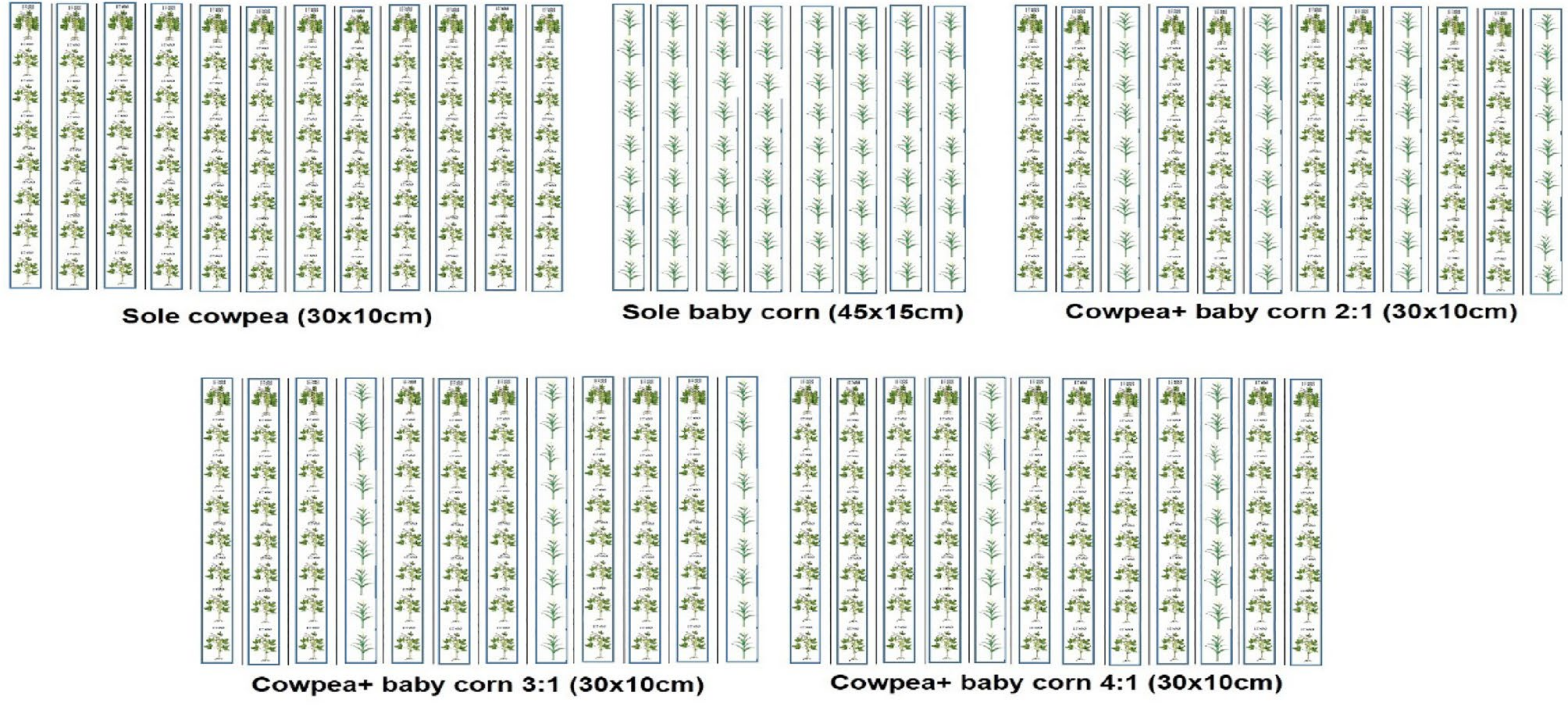
Experimental plot design illustrating sowing pattern of evaluated two sole crops (sole cowpea and sole baby corn) and three intercropping systems (IS) of cowpea and baby corn.

The experimental trials were carried out using a split-split plot design with four replications. In the main plot of the study, there were five intercropping systems: sole cowpea, sole baby corn, cowpea + baby corn as 2:1, cowpea + baby corn as 3:1 and cowpea + baby corn as 4:1 row ratio. Three levels of fertilizer viz*.* 100, 125 and 150% RDF were applied in sub plot and in sub-sub plots, stress mitigating chemicals (0.5% CaCl_2_ and 1.0% KNO_3_) sprayed as foliar application (Table 1). It is essential to emphasize that the comparison exclusively entailed evaluating the effects of the two stress-mitigating chemicals in relation to each other, with no inclusion of a control treatment. These stress-reducing chemicals were applied during the flowering and pod development stages of cowpea. Baby corn (var. G 5414) and cowpea (var. GC 4) were used in the study. In keeping with the particular row arrangements (Fig. 2), cowpea and baby corn seeds that were well mature and healthy were simultaneously sown. Cowpea and baby corn seed rates were 30 and 25 kg/ha, respectively.

**Table 1:** Description of the experimental set up.

Treatment	Treatment short form	Split-split plot design 1	Split-split plot design 2	Split-split plot design 3
Main plot (intercropping system)				
(i) Sole cowpea	C_1_	C_1_	–	C_1_
(ii) Sole baby corn	C_2_	–	C_2_	C_2_
(iii) Cowpea + baby corn (2:1)	C_3_	C_3_	C_3_	C_3_
(iv) Cowpea + baby corn (3:1)	C_4_	C_4_	C_4_	C_4_
(v) Cowpea + baby corn (4:1)	C_5_	C_5_	C_5_	C_5_
Sub plot (fertilizer levels)				
(i) 100% RDF (N_20_ P_40_)	F_1_	F_1_	F_1_	F_1_
(ii) 125% RDF (N_25_ P_50_)	F_2_	F_2_	F_2_	F_2_
(iii) 150% RDF (N_30_ P_60_)	F_3_	F_3_	F_3_	F_3_
Sub-sub plot (stress mitigating chemicals)				
(i) 0.5% CaCl_2_	S_1_	S_1_	S_1_	S_1_
(ii) 1% KNO_3_	S_2_	S_2_	S_2_	S_2_

**Figure 3:**
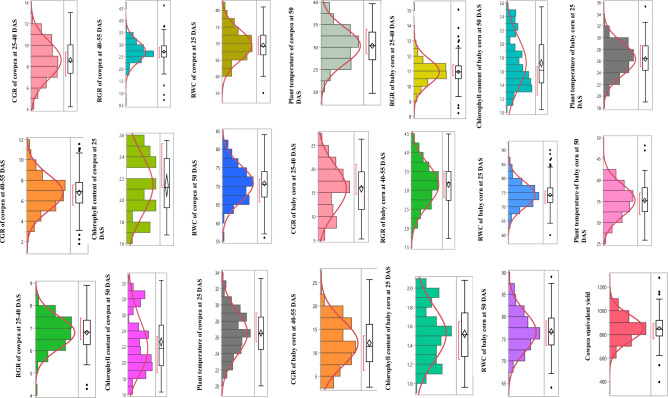
Normal probability plots for different parameters of cowpea and treatment.

The intercropping system was put into replacement series in the first week of April in both the years. The calculated doses for three levels of fertilizer (100%, 125%, and 150% RDF) were applied before sowing in designated plots using urea and single super phosphate (SSP) in the sub plots. During the flowering and pod development stages of cowpea, stress-reducing chemicals, including CaCl_2_ at a rate of 0.5% and KNO_3_, were sprayed using a knapsack sprayer in the sub-sub plots. All experimental methodologies were conducted following relevant guidelines and regulations according to standard protocols. The normal probability plots for all the studied characteristics are shown in Fig. 3. It can be observed that all the characteristics follow a normal distribution except for the chlorophyll content in cowpea and baby corn at both 25 and 50 DAS. Therefore, a log-normal transformation of the chlorophyll observations was performed for further analysis of this particular characteristic.

### Measurements

**Crop growth rate (g/m**^**2**^**/day) by Leopold and Kridermann**^31^. The CGR was computed by using the dry matter of crop at different time periods. The following formula is used to determine the value of CGR:

$${\text{Crop}}\,{\text{growth}}\,{\text{rate}}\,({\text{g}}/{\text{m}}^{2} /{\text{day}}) = \frac{{{\text{w}}_{2} - {\text{w}}_{1} }}{{{\text{t}}_{2} - {\text{t}}_{1} }}$$

where, w_1_ = dry weight at first stage, w_2_ = dry weight at second stage, t_2_−t_1_ = Time interval between two stages in days.

**Relative growth rate (10**^**−2**^** g/g/day) by Dhopte and Manual**^32^. This variable represents the growth rate per unit dry matter. It can be computed by using formula as:

$${\text{Relative}}\,{\text{growth}}\,{\text{rate}}\,({\text{g}}/{\text{g}}/{\text{day}}) = \frac{{{\text{Log}}_{{{\rm e}}} {\text{w}}_{2} - {\text{Log}}_{{\text{e}}} {\text{w}}_{1} }}{{{\text{t}}_{2} - {\text{t}}_{1} }}$$

**Chlorophyll content (mg/g fresh weight) by Hiscox and Israelstam**^33^. The leaf sample for both crops was taken by taking five healthy fully opened leaves from each plant 25 and 50 DAS (days after sowing). The leaves were finely crushed and 20 mg of sample was taken. A sample of 20 mg was collected from the finely crushed leaves. The pigments from the prepared samples were extracted using the dimethyl sulphoxide DMSO extraction method, by suspending the leaves samples in 5 ml of dimethyl sulphoxide (DMSO) and thereafter, incubating it at 60 °C for about one hour in a pre-heated hot air oven. Each extract’s absorbance was measured using a spectrophotometer at a wavelength of 652 nm. The following equations were used to calculate the total content:

$${\text{Total}}\;{\text{chlorophyll}} = \frac{{{\text{A}}_{{(652)}} \times 29 \times {\text{Total}}\;{\text{volume}}\left( {{\text{ml}}} \right)}}{{\upalpha \times 1000 \times {\text{weight}}\;{\text{of}}\;{\text{sample}}\left( {\text{g}} \right)}}$$

where, α is the path length = 1 cm; 29 is constant.

**Relative water content (%)**. A plant sample was acquired by selecting five healthy plants from each plot of both crops independently to calculate the relative water content. The plants were then weighed right away to get a current weight. After that, the plants were floated in distilled water until fully turgid, then weighed to determine the turgid weight. Finally, the samples of plants were placed for 48 h at 60 °C in a preheated oven. The samples were placed in oven until they reached a homogeneous dry weight. Finally, RWC was calculated by the following formula-

$${\text{Relative}}\,{\text{Water}}\,{\text{Content}}(\% ) = \frac{{{\text{f.w.}} - {\text{d.w.}}}}{{\text{t.w.}} - {\text{d.w.}}} \times 100$$

where, f.w. = Fresh sample weight (g), d.w. = Dry sample weight (g), t.w. = Turgid sample weight (g).

**Plant temperature (**°C**)**. Plant temperature was recorded at 2 pm 25 and 50 DAS by infrared thermometer. To record the plant temperature 5 random plants were selected of both crops and temperature was taken from upper, middle and lower portion of plant and averaged.

**Cowpea equivalent yield**. It is used to make comparison of crops on the basis of gross return. The yield of different intercrops was converted into equivalent yield of any one crop based on the price of the produce. The baby corn yield was converted into equivalent yield of cowpea by following formula^34^:

$${\text{CEY}}=\frac{Yield\, of\, babycorn \left(\frac{kg}{ha}\right)X Price\, of\, babycorn (\frac{Rs.}{kg}) }{Price\, of\, cowpea (\frac{Rs.}{kg})}$$

### Statistical data analysis

Utilizing SAS version 9.4, the experimental data were subjected to split-split plot design analysis of variance using various treatments with intercropping system (sole cowpea, sole baby corn, cowpea + baby corn as 2:1, cowpea + baby corn as 3:1, and cowpea + baby corn as 4:1 row ratio) as main factors, fertilizer levels (100, 125 and 150% RDF) as sub factors and two stress mitigating chemicals (0.5% CaCl_2_ and 1% KNO_3_) as sub-sub factors. To distinguish the statistically importance of the differences between the mean values, the Duncan’s Multiple Range Test at probability 5% was applied.

## Results

### Significance of tests of intercropping systems, levels of fertilizer and foliar application of stress mitigating chemicals

**Figure 4:**
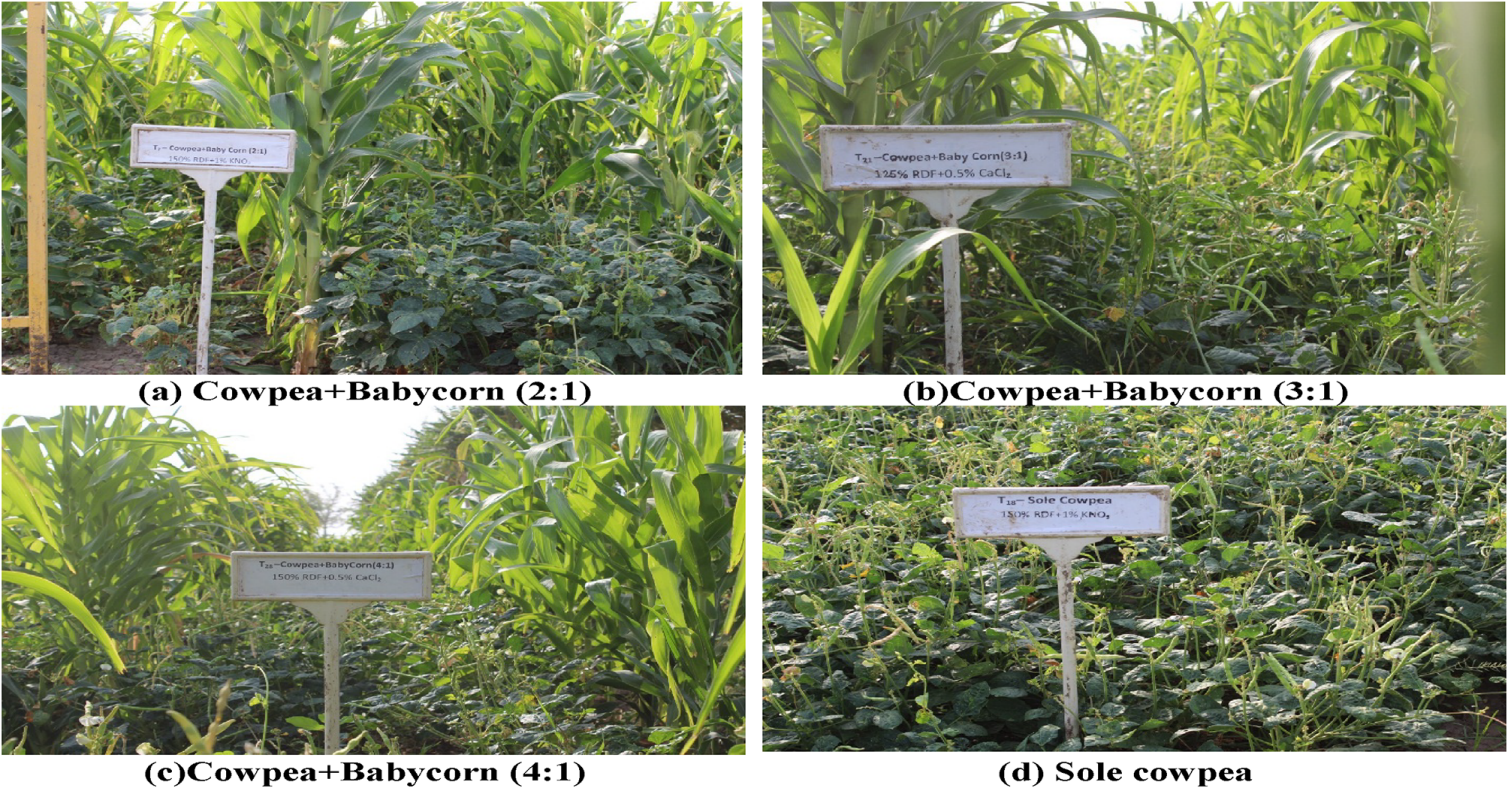
Pictorial view of different treatments (**a**) Cowpea + Babycorn (2:1), (**b**) Cowpea + Babycorn (3:1), (**c**) Cowpea + Babycorn (4:1), (**d**) Sole cowpea in field at experimental sites.

The analysis of variance (ANOVA) tables for Tables 2, 3 and 4, conducted on pooled data from two years, demonstrates that intercropping systems, levels of fertilizer, and two stress mitigation chemicals in relation to each other, without control treatment had significant impacts on various factors, including CGR, RGR and chlorophyll content in both cowpea and baby corn. Additionally, intercropping and the application of stress mitigation chemicals had a notable influence on RWC and plant temperature. Pictorial view of three intercropping systems and sole cowpea has been given in Fig. 4. The outcomes derived from experimental supplements 20a and 20b present the results of the ANOVA conducted on cowpea and baby corn across various variables. The findings suggest that, apart from CGR 25 and 50 DAS, chlorophyll content 25 DAS, and seed yield of cowpea, the majority of variables did not exhibit statistically significant differences between the respective years. However, a statistically significant divergence was observed in RWC 25 DAS, as well as chlorophyll content 25 and 50 DAS for baby corn.

**Table 2:** Analysis of variance (mean sum of squares) for different parameters at different stages and seed yield of summer cowpea.

Source	DF	CGR		RGR		Chlorophyll content		RWC		Plant temperature		Seed yield
		25–40 DAS	40–55 DAS	25–40 DAS	40–55 DAS	25 DAS	50 DAS	25 DAS	50 DAS	25 DAS	50 DAS	
REP	3	0.569	0.148	0.1570	0.0943	0.0090	0.0030	17.531	13.459	7.231	15.759*	4502.06
C	3	29.196*	41.31*	1.4897*	0.8489*	2.6646*	3.8256*	17.499	143.191*	2.696	166.397*	819302.78*
Error(a)	9	0.553	0.678	0.2212	0.1844	0.0045	0.0010	17.762	10.436	3.613	1.868	20972.08
F	2	39.643*	28.822*	2.5107*	0.1491	0.1117*	0.1002*	10.877	23.137	9.875	2.792	153074.02*
C*F	6	0.306	0.994	0.1696	0.0963	0.0037	0.0009	1.458	1.626	3.772	8.579	4088.09
Error(b)	24	0.489	0.84	0.3014	0.1586	0.0076	0.0057*	17.779	14.138	5.79	7.417	75314.60
S	1	50.918*	21.236*	13.2021*	0.1471	0.0060	0.1441	55.434	244.641*	12.76	171.775*	27034.59*
C*S	3	0.032	0.774	0.0322	0.1759	0.0019	0.0013	6.373	6.69	1.452	0.404	133.47
F*S	2	2.175*	1.347	0.3810	0.3301	0.0016	0.0006	0.513	7.348	7.528	4.162	4099.27
C*F*S	6	0.264	1.008	0.1173	0.1547	0.0010	0.0032	0.38	6.077	6.535	1.574	11238.17
Error(c)	36	0.278	0.579	0.1405	0.1161	0.0055	0.0076	29.069	21.111	7.162	8.586	34423.38

*indicates significant at 5%; REP, Replication; C, Intercropping system; F, Fertilizer; S, Stress mitigating chemicals.

**Table 3:** Analysis of variance (mean sum of squares) for different parameters at different stages and cob yield of summer baby corn.

Source	DF	CGR		RGR		Chlorophyll content		RWC		Plant temperature		Cob yield
		25–40 DAS	40–55 DAS	25–40 DAS	40–55 DAS	25 DAS	50 DAS	25 DAS	50 DAS	25 DAS	50 DAS	
REP	3	1.568	4.003	1.1386	0.4340	0.0018	0.0055	10.062	29.645	8.06	24.548	10.29
C	3	659.715*	484.579*	1.6264	0.5227	2.3842*	4.0256*	11.134	7.824	2.506	151.952*	12065.37*
Error(a)	9	0.907	4.965	0.4536	0.3703	0.0092	0.0026	24.855	11.1	3.009	9.548	15.73
F	2	83.124*	194.722*	0.1403	2.3307*	0.0599*	0.0682*	16.525	8.745	0.015	3.156	144.84*
C*F	6	1.532	7.981*	0.1401	0.4141	0.001	0.0114*	9.233	3.313	3.984	6.002	11.21
Error(b)	24	2.251	2.354	0.7886	0.2235	0.0035*	0.0044	14.422	18.814	8.127	6.929	47.66
S	1	64.317*	74.637*	6.2549*	0.9552*	0.0004	0.0213*	43.001	255.552*	12.028	122.967*	12.99*
C*S	3	3.450*	2.564	0.2113	0.6919*	0.0043	0.0470*	16.409	4.765	0.828	2.707	4.69
F*S	2	1.365	1.931	0.1346	0.0002	0.0036	0.0001	16.584	6.927	9.079	2.123	1.92
C*F*S	6	0.989	1.959	0.3333	0.1014	0.0007	0.0037	2.392	5.641	5.997	4.011	2.32
Error(c)	36	1.202	2.81	0.5253	0.1862	0.0031	0.0048	26.837	29.646	7.315	8.928	69.71

*indicates significant at 5%; REP, Replication; C, Intercropping system; F, Fertilizer; S, Stress mitigating chemicals.

**Table 4:** Analysis of variance for cowpea equivalent yield.

Source	DF	Sum of squares	Mean square	F-ratio	Significant
REP	3	2685.700	895.2333	0.3704	NS
C	4	573184.283	143296.0708	59.2866	*
Error(a)	12	29004.050	2417.0042		
F	2	247800.350	123900.1750	36.6086	*
C*F	8	41878.567	5234.8208	1.5467	NS
Error(b)	30	101533.750	3384.4583		
S	1	48561.633	48561.6333	24.3695	*
C*S	4	4058.617	1014.6542	0.5092	NS
F*S	2	4711.517	2355.7583	1.1822	NS
C*F*S	8	10906.733	1363.3417	0.6842	NS
Error(c)	45	89672.500	1992.7222		
Total	119	1153997.7000			

REP, Replication; C, Intercropping system; F, Fertilizer; S, Stress mitigating chemicals.

*Significant at 5% (level of significance opted by user); NS, Non-Significant; *p*-value < 0.05, Significant at 5%; *p*-value < 0.01, Significant at 1%.

### Cowpea (split-split plot design 1)

#### Effect of intercropping (main plot)

**Figure 5:**
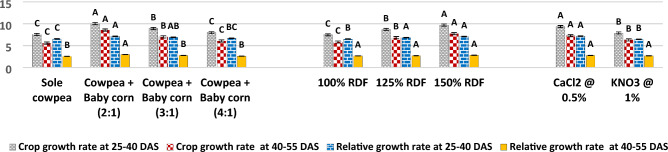
CGR (g/m^2^/day) and RGR (g/g/day) of cowpea as affected by different intercropping systems, fertilizer levels and stress mitigating chemicals.

**Figure 6:**
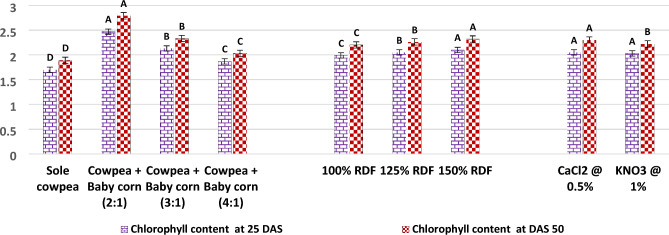
Chlorophyll content (mg/g) of cowpea as affected by different intercropping systems, fertilizer levels and stress mitigating chemicals.

**Figure 7:**
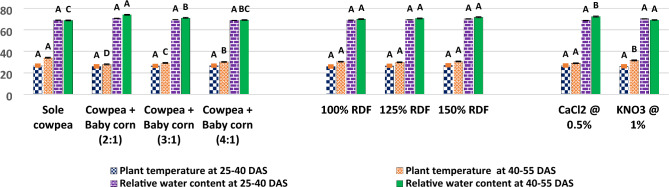
Plant temperature (°C) and relative water content (%) cowpea as affected by different intercropping systems, fertilizer levels and stress mitigating chemicals.

In the current study, as illustrated in Fig. 5, it was observed that the 2:1 row ratio of cowpea + baby corn exhibited the highest CGR of cowpea during the 25–40 DAS period, with a value of 10.03 g/m^2^/day. Similarly, during the 40–55 DAS period, the CGR remained notably high with 8.55 g/m^2^/day. These findings were found to be statistically significant when compared to the CGR observed in sole cowpea and other row ratios, namely, the 3:1 and 4:1 row ratio of cowpea and baby corn. Results further showed that RGR of cowpea was also highest with 2:1 row ratio of cowpea + baby corn at 25–40 (7.11 10^**−**2^ g/g/day) and 40–55 DAS (2.96 10^**−**2^ g/g/day) over rest of treatments. Turning to the analysis of chlorophyll content and RWC in cowpea, the results from Figs. 6 and 7 in the current study revealed that these parameters showed significant increases in all intercropping systems as compared to sole cowpea cultivation. Among the various intercropping systems, the 2:1 row ratio notably yielded higher chlorophyll content, measuring 2.47 and 2.79 mg/g 25 and 50 DAS, respectively. 50 DAS, RWC in cowpea reached at 74.08% when cultivated in the same 2:1 row ratio, contrasting with the levels observed in sole crop cultivation and other row ratios of cowpea + baby corn. 25 DAS, RWC remained unaffected and showed no significant variation, irrespective of the intercropping system employed. Regarding plant temperature, different intercropping systems demonstrated a significant impact on lowering the temperature of cowpea plants 50 DAS.

The highest recorded plant temperature was observed in the sole cowpea cultivation, registering at 34 °C. In contrast, the lowest temperature was recorded in the intercropping system involving cowpea + baby corn with a 2:1 row ratio. Specifically, in the 2:1 cowpea + baby corn intercropping system, the plant temperature of cowpea was found to be lower than that of sole cowpea, cowpea + baby corn (3:1), and cowpea + baby corn (4:1), with differences of 6.1 °C, 1.23 °C, and 2.31 °C, respectively.

#### Effect of fertilizer levels (sub plot)

As shown in Fig. 5, there is a conspicuous trend where increasing levels of fertilizer have a substantial impact on both the CGR and RGR of cowpea. As fertilizer levels gradually escalated, particularly up to 150% of the RDF, there was a pronounced enhancement in CGR. During the growth phases of 25–40 and 40–55 DAS, the CGR demonstrated a significant surge, reaching 9.69 g/m^2^/day and 7.73 g/m^2^/day, respectively. The RGR of cowpea mirrored this trend, registering values of 7.07 10^**−**2^ g/g/day during the 25–40 DAS period and 2.79 10^**−**2^ g/g/day during the 40–55 DAS period, clearly outperforming the lower levels.

The experimental results from Figs. 6 and 7 unveil an intriguing dynamic between fertilizer levels and cowpea physiology. At fertilizer level of 150% RDF, there was a marked and statistically significant increase in the chlorophyll content within the leaves of cowpea, exhibiting values of 2.10 g/mg 25 DAS and 2.32 g/mg 50 DAS. Conversely, the RWC of summer cowpea displayed a resilient stability across various levels of fertilizer throughout the entirety of the investigation. Intriguingly, the data presented in Fig. 7 indicate that none of the examined levels of fertilizer brought about significant variations in the plant temperature of summer cowpea at any of the growth stages considered.

#### Effect of stress mitigating chemicals (sub-sub plot)

The data presented in Fig. 5 indicates that among the two tested stress-mitigating chemicals, application of 0.5% CaCl_2_ during the flowering and pod development stages of cowpea led to significantly higher CGR during 25–40 DAS period (9.34 g/m^2^/day) and 40–55 DAS period (7.26 g/m^2^/day). Additionally, RGR during 25–40 DAS period showed a notable increase, reaching 7.17 10^**−**2^ g/g/day. However, during the 40–55 DAS period, the RGR of cowpea remained unaffected by the foliar application of 0.5% CaCl_2_ and 1.0% KNO_3_.

The results presented in Figs. 6 and 7 also reveal that the foliar spray of 0.5% CaCl_2_ and 1.0% KNO_3_ did not lead to statistically significant changes in the chlorophyll content and RWC of cowpea 25 DAS. However, 50 DAS, the application of 0.5% CaCl_2_ during the flowering and pod development stages significantly increased the chlorophyll content to 2.30 mg/g and the RWC to 72.35%, when compared to the effects of 1.0% KNO_3_.

Furthermore, the data from Fig. 6 indicates that the use of stress mitigating chemicals did not result in any significant variation in the plant temperature of summer cowpea 25 DAS. However, 50 DAS, the application of 0.5% CaCl_2_ achieved the lowest recorded canopy temperature for cowpea, measuring 28.97 °C.

### Baby corn (split-split plot design 2)

#### Effect of intercropping (main plot)

**Figure 8:**
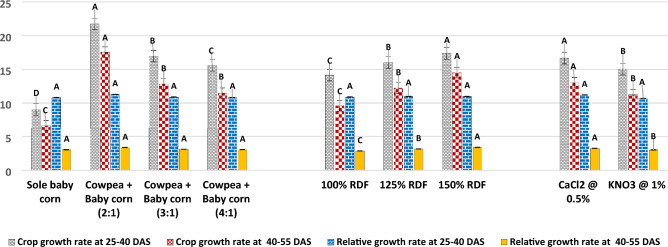
CGR (g/m^2^/day) and RGR (g/g/day) of baby corn as affected by different intercropping systems, fertilizer levels and stress mitigating chemicals.

The experimental results depicted in Fig. 8 indicate that both the CGR and RGR of baby corn exhibited significant improvement when cultivated in a 2:1 row ratio of cowpea + baby corn during the 25–40 DAS period, with CGR at 21.74 g/m^2^/day and RGR at 11.3 × 10^**−**2^ g/g/day. Furthermore, during the 40–55 DAS period, the same 2:1 row ratio of cowpea + baby corn continued to outperform, with CGR at 17.55 g/m^2^/day and RGR at 3.37 × 10^**−**2^ g/g/day. These results were notably superior to those achieved in both sole baby corn cultivation and other intercropping row ratios.

**Figure 9:**
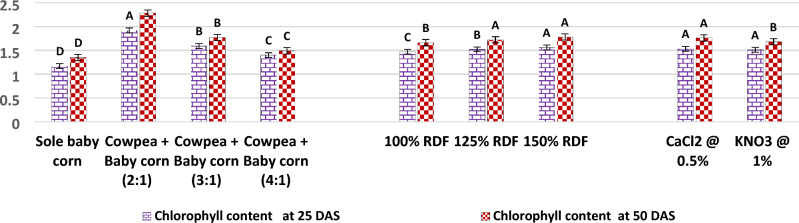
Chlorophyll content (mg/g) of baby corn as affected by different intercropping systems, fertilizer levels and stress mitigating chemicals.

**Figure 10:**
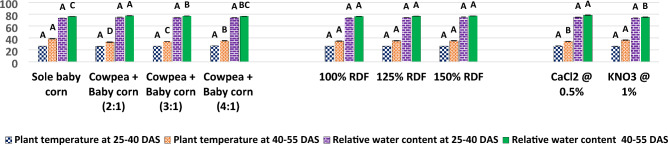
Plant temperature (°C) and relative water content (%) of baby corn as affected by different intercropping systems, fertilizer levels and stress mitigating chemicals.

Additionally, the findings from Figs. 9 and 10 underscore the significance of the 2:1 row ratio of cowpea + baby corn, revealing substantial variations in the chlorophyll content of baby corn. Specifically, chlorophyll content reached 1.92 mg/g at 25 DAS and 2.38 mg/g at 50 DAS when compared to sole baby corn, 3:1 and 4:1 row ratio of cowpea + baby corn. In contrast, RWC of baby corn at both 25 and 50 DAS displayed statistically non-significant variations across all intercropping systems and sole cropping.

Notably, the plant temperature of baby corn, as shown in Fig. 10, exhibited the highest reading in sole baby corn at 38.76 °C, while the lowest temperature of 33.01 °C was observed in the 2:1 row ratio of cowpea + baby corn. The variation in plant temperature for the 2:1 row ratio was consistently lower, with deviations of 5.75 °C, 0.97 °C, and 2.18 °C, as compared to sole baby corn, 3:1, and 4:1 row ratio of cowpea + baby corn, respectively.

#### Effect of fertilizer levels (sub plot)

In the current study, both CGR and RGR were significantly affected by varying fertilizer levels at different growth stages. Notably, when fertilizer level equivalent to 150% of the RDF was applied, it led to a substantial increase in CGR. Specifically, this increase amounted to 22.7% and 51.8% compared to the use of 100% RDF, and 8.3% and 18.6% compared to 125% RDF during the 25–40 DAS and 40–55 DAS, respectively (Fig. 8).

Upon an examination of the recorded values (Figs. 9 and 10), it is evident that the application of the 150% RDF resulted in significantly higher chlorophyll content in baby corn. 25 DAS, the chlorophyll content reached 1.56 mg/g, and 50 DAS, it increased to 1.78 mg/g, representing substantial increases over the levels observed with previous fertilization levels. These increases amounted to 5.9% and 2.5% 25 DAS and 6.8% and 2.9% 50 DAS when compared to the use of 100% and 125% RDF, respectively. In contrast, the RWC of baby corn was not significantly influenced by different levels of fertilizer at any of the growth stages.

The data presented in Fig. 10 indicate that none of the applied levels of fertilizer brought about significant variations in the plant temperature of summer baby corn at any of the growth stages.

#### Effect of stress mitigating chemicals (sub-sub plot)

In the sub-sub plots, a comparative analysis was conducted between two stress mitigating chemicals. The findings from Fig. 8 indicate that application of 0.5% CaCl_2_ spray during the flowering and pod development stages led to significantly higher CGR during 25–40 DAS with a value of 16.66 g/m^2^/day and during 40–55 DAS with a value of 13.00 g/m^2^/day. Similarly, RGR was significantly increased during 25–40 DAS (11.2 × 10^**−**2^ g/g/day) and 40–55 DAS (3.25 × 10^**−**2^ g/g/day) for baby corn compared to the use of 1.0% KNO_3_.

Data presented in Figs. 9 and 10 reveal that chlorophyll content and RWC remained statistically unchanged when exposed to various stress mitigating chemicals at 25 DAS. However, 50 DAS, the application of 0.5% CaCl_2_ resulted in significantly higher chlorophyll content (1.78 mg/g) and RWC (78.28%) when compared to the use of 1.0% KNO_3_ under pooled mean.

Results from Fig. 10 demonstrate that the application of stress mitigating chemicals did not produce any significant variation in the canopy temperature of baby corn at 25 DAS. Nonetheless, 50 DAS, the spray of 0.5% CaCl_2_ resulted in a significantly lower plant temperature of 34.10 °C, in contrast to the 1.0% KNO_3_ treatment with a temperature of 36.37 °C.

### Cowpea-equivalent yield (split-split plot design 3)

#### Effect of intercropping (main plot)

**Figure 11:**
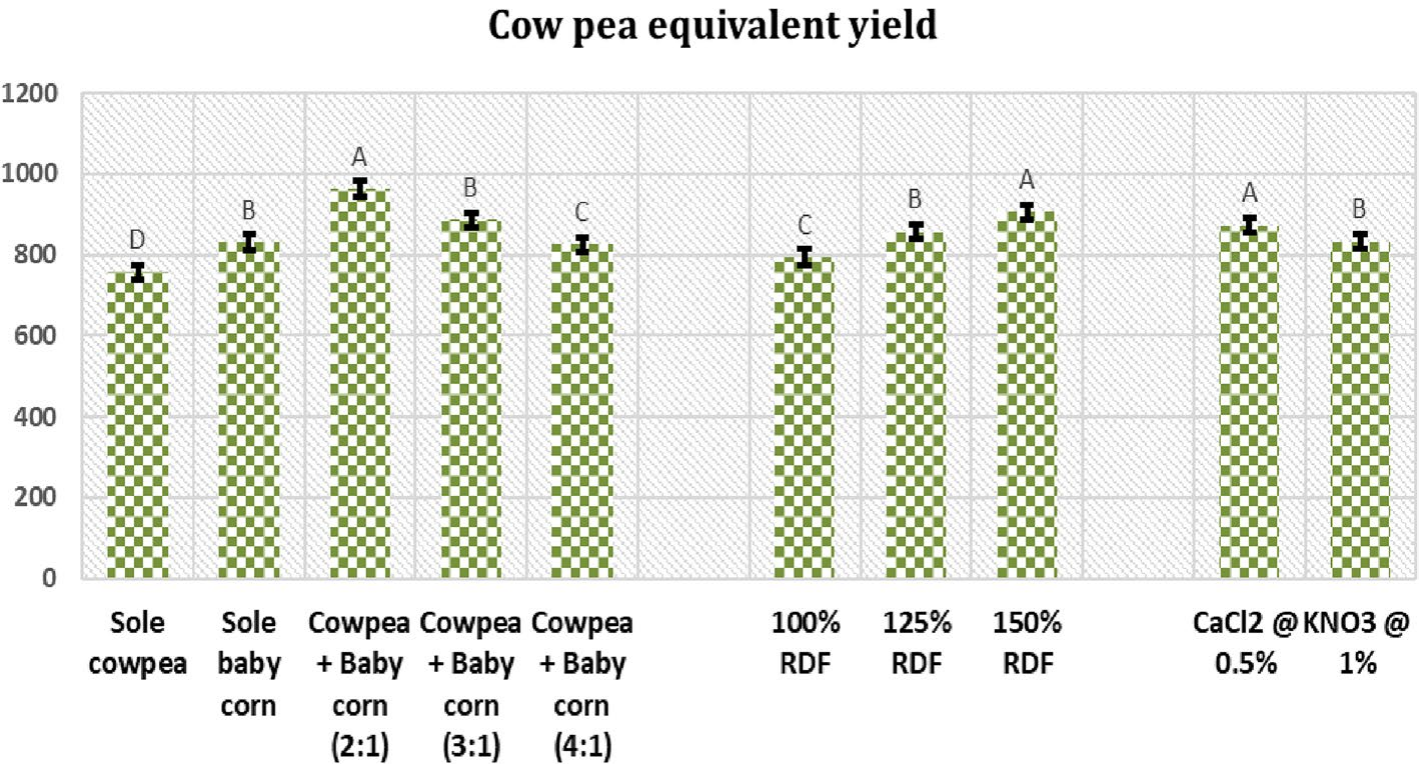
Cowpea equivalent yield (kg/ha) as affected by different intercropping systems, fertilizer levels and stress mitigating chemicals. *Note* Treatments having same alphabetical letters in Figs. 5, 6, 7, 8, 9, 10 and 11 within different intercropping systems (C), fertilizer levels (F) and stress mitigating chemicals (S) indicates that treatments do not differ significantly from each other within “C”, “F” and “S” respectively.

In the current study, the results presented in Fig. 11 demonstrated that intercropping cowpea + baby corn in a 2:1 row ratio resulted in a significantly higher cowpea-equivalent yield of 963 kg/ha. This yield was 27.38% higher than sole cowpea, 16.02% higher than sole baby corn, and 16.73% and 8.81% higher than the 4:1 and 3:1 row ratios, respectively.

#### Effect of fertilizer levels (sub plot)

The maximum cowpea-equivalent yield of 906 kg/ha was recorded with the application of 150% RDF, as shown in Fig. 11. This represented a percentage increase of 14.1% and 5.8% over the use of 125% and 100% RDF, respectively.

#### Effect of stress mitigating chemicals (sub-sub plot)

The comparative assessment of two stress mitigating chemicals in Fig. 11 reveals that the foliar application of 0.5% CaCl_2_ significantly enhanced the cowpea equivalent yield to 872 kg/ha, which was a substantial improvement compared to the use of 1.0% KNO_3_. Specifically, the cowpea equivalent yield increased by 4.81% due to the application of 0.5% CaCl_2_ over the use of 1.0% KNO_3_. This suggests that CaCl_2_ at a concentration of 0.5% was more effective in increasing cowpea yield compared to KNO_3_ at 1.0%.

#### Interactive effect of intercropping, fertilizer levels and stress mitigating chemical

The results of the study indicate that the interaction between intercropping, fertilizer levels, and stress mitigating chemicals did not demonstrate statistical significance concerning various parameters measured across different growth stages. These parameters encompassed CGR, RGR, Chlorophyll content, RWC and canopy temperature for both cowpea and baby corn, as well as cowpea equivalent yield. For more detailed information on the interaction effects of these factors on the parameters in question, refer to “supplementary Table 11, 13, and 15 in the supplementary file”.

### Cowpea (split-split plot design 1)

Supplement 11 provide a more nuanced view of how different treatment combinations influenced the measured parameters of cowpea. Although statistical significance was not achieved in the overall analysis, the nuanced examination of various parameters of cowpea revealed promising trends. In the realm of CGR in cowpea, it was observed that during 25–40 DAS, a noteworthy CGR of 12.06 g/m^2^/day was recorded. This was followed by a slightly reduced, yet substantial, CGR of 10.72 g/m^2^/day during 40–55 DAS. These findings suggest that the treatment combination, intercropping of cowpea + baby corn in 2:1 row ratio and fertilizer levels set at 150% RDF, along with the application of 0.5% CaCl_2_ during the flowering and pod development stages of cowpea, played a discernible role in enhancing the growth rate of cowpea at both early and later stages over all the treatment combination. RGR of cowpea also exhibited notable trends with the same treatment combination. 25–40 DAS, RGR was measured at 7.71 × 10^**−**2^ g/g/day, indicating a higher relative growth rate during this phase. Subsequently, during 40–55 DAS, the RGR remained respectable at 3.17 × 10^**−**2^. These figures further underscore the potential benefits of the specified treatment combination in promoting robust growth and development of cowpea plants. Furthermore, the study examined additional critical parameters, including chlorophyll content, RWC and plant temperature. In the treatment combination involving intercropping of cowpea + baby corn with a 2:1 row ratio, fertilization at 150% RDF and the application of 0.5% CaCl_2_, chlorophyll content exhibited values of 2.52 mg/g 25 DAS and 2.89 mg/g 50 DAS, which were the highest among all the treatment combinations. Compared to other treatment combinations involving intercropping, varying levels of fertilizer, and stress mitigation chemicals, both RWC and plant temperature 50 DAS also displayed positive results with this particular combination. Plant temperature was notably recorded at 26.63 °C 50 DAS, representing the lowest value among all treatment combinations, while highest RWC measured at 76.63% 50 DAS. The treatment combination involving a 2:1 row ratio, level of fertilizer at 150% RDF, and the application of 0.5% CaCl_2_ during specific growth stages appeared to positively impact CGR, RGR, chlorophyll content, RWC and plant temperature.

While examining various growth parameters, the study sought to understand the combined impact of intercropping, different fertilizer level, and the application of stress-mitigating chemicals on cowpea yield. Overall, the results did not reveal statistically significant differences. However, when analyzing cowpea seed yield, a noteworthy trend emerged. In the case of the sole cowpea field fertilized with 150% RDF and treated with a foliar spray of a 0.5% CaCl2 solution, a slight increase (810 kg/ha) was observed. This particular combination demonstrated marginally higher cowpea seed yield.

### Baby corn (split-split plot design 2)

Within the confines of Supplement 13, it becomes evident that the intricate interplay between intercropping, levels of fertilizer, and the application of stress mitigating chemicals did not yield statistically significant differences in the measured parameters for baby corn. However, a closer examination reveals intriguing trends, where specific treatment combinations demonstrated marginally higher values.

Notably, the CGR during 25–40 DAS showcased an increasing trend, recording a value of 24.64 g/m^2^/day. This observation suggests an improving growth trajectory during the initial growth phase for baby corn. Furthermore, during 40–55 DAS, the CGR remained notably substantial at 20.8 g/m^2^/day, albeit with a slight reduction. Turning attention to the RGR, the results were equally compelling. During 25–40 DAS, the RGR was measured 11.53 × 10^**−**2^ g/g/day, indicating a period of rapid relative growth during the early stages of baby corn development. Subsequently, during 40–55 DAS, the RGR saw a decline to 3.5 × 10^**−**2^ g/g/day. This shift suggests a transition to a slightly slower but nonetheless noteworthy relative growth rate as the crop progressed. Chlorophyll content, displayed a value of 1.97 mg/g 25 DAS. However, 50 DAS, the chlorophyll content showed an increase, reaching 2.37 mg/g. This augmentation in chlorophyll levels during the later growth stage underscores the potential benefits of the specified treatment combinations in enhancing photosynthesis and overall vitality in baby corn plants. Interestingly, these higher values in CGR, RGR, and chlorophyll content were consistently associated with the treatment combination of a 2:1 row ratio, fertilization at 150% RDF, and the application of CaCl_2_ over other treatment combination.

Moreover, the RWC held steadfast with prior results, with a particularly noteworthy value of 79.93% 50 DAS. This value implies that the specific treatment combination involving a 2:1 row ratio, fertilization at 125% of the RDF, and the application of 0.5% CaCl_2_ had a marginally greater impact on maintaining optimal water content during this specific growth stage for baby corn as compare to other treatment combination. Plant temperature 50 DAS was also lowest in the same treatment combination, measuring 31.14 °C.

Further, collective influence of intercropping, varying fertilizer level, and stress-mitigating chemicals on baby corn yield. Overall, the results did not exhibit statistically significant differences. However, a slight increase (4329 kg/ha) was observed in the sole baby corn field when fertilized with 150% RDF and subjected to a foliar spray of a 0.5% CaCl_2_ solution. This particular combination demonstrated a marginally higher baby corn yield.

### Cowpea-equivalent yield (split-split plot design 3)

Results from Supplement 15 indicate that among the tested treatment combinations, the highest cowpea equivalent yield was achieved with intercropping of cowpea + baby corn in 2:1 row ratio, fertilized with 150% RDF and application of 0.5% CaCl_2_ spraying during the flowering and pod development stages of cowpea. This treatment resulted in a cowpea equivalent yield of 1083 kg/ha and it was followed by the treatment combination involving intercropping with the 2:1 row ratio of cowpea + baby corn, 125% RD, and 0.5% CaCl_2_ spray. However, the results of interaction were found statistically non-significant in respect to cowpea equivalent yield.

## Discussion

### Intercropping

Since CGR and RGR are determined by the accumulation of dry matter per day, the increase in CGR and RGR observed in cowpea and baby corn in a row ratio of 2:1 is likely attributed to a higher production of dry-matter within this row configuration. The enhanced dry matter production in intercropping can be attributed to a more efficient distribution of light throughout the lower canopy, as well as the symbiotic nitrogen fixation facilitated by cowpea when intercropped with baby corn. Intercropping often relies on the principle of complementarity, where different crops have varying resource requirements and growth patterns that benefit each other. In a 2:1 ratio, the potential for complementarity between the two crops is better realized. For example, if cowpea (a legume) is the dominant crop, it can fix nitrogen, benefiting the baby corn. In broader ratios, the secondary crop’s contribution to complementarity diminishes as its presence becomes less significant. In 2:1 ratio, the microclimate within the intercropped area is more balanced. The dominant crop may provide some shading, reducing weed growth and moderating temperature and humidity, which can be beneficial to both crops. These findings confirm the results of Addo-quaye et al.^35^ and Telkar et al.^36^. The observed beneficial effects of intercropping on plant temperature and RWC in cowpea and baby corn can be attributed to several underlying mechanisms. The shading provided by the taller baby corn plants to the cowpea canopy plays a pivotal role in reducing the heat load experienced by the crops. This shading effect, particularly in the scorching summer conditions, contributes to lower plant temperatures and, in turn, promotes higher RWC levels in the summer-grown cowpea. Another noteworthy aspect contributing to the favorable microclimate within intercropping systems is the complete interplanting of cowpea within the baby corn rows^37^. Turning to chlorophyll content, a key indicator of photosynthetic activity, it is noteworthy that cowpea exhibits significantly higher chlorophyll levels in intercropping systems compared to sole cropping. This phenomenon can be attributed to the adaptive response of cowpea to the shaded environment within intercropping, which prompts the plant to produce a greater quantity of chlorophyll in its leaves^38^. This enhanced chlorophyll production leads to improved photosynthetic efficiency and, consequently, higher growth rates and biomass accumulation. Similar findings have been reported in related research, such as Pandey’s^39^ investigation into maize and soybean intercropping systems, where the provision of shade resulted in elevated chlorophyll content.

In the case of baby corn, a significant increase in chlorophyll content is observed across all intercropping systems compared to sole baby corn cultivation. This boost in chlorophyll levels can be primarily attributed to the presence of intercropped cowpea, which not only contributes to cooling but also provides atmospheric nitrogen fixation benefits to baby corn. Nitrogen is a key element required for chlorophyll synthesis, and the availability of biologically fixed nitrogen from cowpea can stimulate chlorophyll production and enhance the photosynthetic capacity of baby corn. Comparable outcomes have been documented in other intercropping scenarios, where legume crops have been found to positively influence the chlorophyll content of associated crops^40,41^.

Furthermore, the higher cowpea equivalent yield observed in the 2:1 row ratio in intercropping systems can be attributed to a multitude of advantages and improvements derived from intercropping practices. The research conducted during the summer season, characterized by high temperatures, provides valuable insights into the complex interplay of environmental factors, crop interactions, and resource dynamics that influence crop performance in different row ratios. As compare to border row ratios, in 2:1 row ratio, baby corn plants are densely planted in close proximity to each other. This spatial arrangement has notable implications for the microclimate within the crop canopy. Specifically, it can reduce the heat load on cowpea plants. The closely spaced baby corn plants may act as a natural shading mechanism, mitigating the intensity of direct sunlight and thermal stress on neighboring cowpea plants. Consequently, the cowpea plants in this ratio could experience a more favorable temperature environment for growth, potentially contributing to higher yields. Moreover, it is important to consider the contrasting N requirements of the two crops. Baby corn has a higher N requirement compared to cowpea. However, cowpea possesses the unique ability to fix atmospheric nitrogen into a form usable by plants. This creates a potential complementary relationship between the two crops. In the 2:1 row ratio, cowpea may supply fixed nitrogen to adjacent baby corn plants, effectively addressing their nutrient needs. This mutualistic interaction may enhance the overall health and vigor of both crops, resulting in improved yields. Furthermore, the contribution of baby corn to soil improvement should not be overlooked. Through root exudates, organic matter decomposition, and root structure, baby corn can collectively contribute to soil health. This enhanced soil condition, particularly in the 2:1 row ratio with higher baby corn density, may provide a more conducive environment for crop growth. Improved soil quality can influence nutrient availability, moisture retention, and overall plant health, potentially explaining the higher yields observed^15^. Such findings are consistent with prior studies by Devi and Singh^42^ and Bijarnia et al. (2021)^31^, which have consistently demonstrated the economic and agronomic benefits of intercropping strategies.

### Fertilizer levels

An increase levels of fertilizer up to 150% RDF was showed a marked impact on CGR and RGR of both cowpea and baby corn over the lower levels of fertilizer (100 and 125% RDF). This phenomenon can be ascribed to the progressive increments in levels of fertilizer, which in turn contributed to a substantial enhancement in the production of dry matter, subsequently elevating the CGR and RGR values for both of the crops. Chlorophyll content in the leaves of cowpea and baby corn also exhibited a significant increase at the 150% RDF level. This elevation can be directly linked to the role of nitrogen as a vital component in the synthesis of chlorophyll. As the nutrient levels, particularly nitrogen, increased, so did the chlorophyll content, reinforcing the positive correlation between nutrient availability and chlorophyll production. These results confirm the findings of Ali et al.^43^ and Bute et al.^44^. The results also revealed that, the fertilizer level of 150% RDF, specifically involving nitrogen-phosphorus combination of N_30_P_60_, resulted in a statistically significant cowpea-equivalent yield of 906 kg/ha, surpassing the yields obtained at the lower levels of fertilizer. This enhancement in yield can be attributed to the overall higher productivity of cowpea and baby corn intercropping at this particular level of nitrogen and phosphorus.

### Stress-mitigating chemicals

The application of 0.5% CaCl_2_, during the flowering and pod-development stage of cowpea resulted in significantly greater CGR during all growth stages, as well as higher RGR during 25–40 DAS than the other treatments. In the case of baby corn, the use of 0.5% CaCl_2_ also led to significantly higher CGR and RGR values throughout all the growth stages. These enhanced CGR and RGR values in both cowpea and baby corn can be attributed to the increased dry-matter content associated with the application of 0.5% CaCl_2_. The foliar application of CaCl_2_ at this concentration likely contributed to improved photosynthetic efficiency, which, in turn, led to higher dry-matter accumulation. Further, the availability of Ca^2+^ ions induced an increase in chlorophyll content and photosynthesis, resulting in taller plant growth, in accordance with previous research findings (Wahid et al., 2007)^9^. These results support the findings of Mohamed and Basalah^45^ and Youssef et al.^46^.

The study showed that, spraying 0.5% CaCl_2_ at 50 DAS significantly increased the chlorophyll content of both cowpea and baby corn compared to the application of 1.0% KNO_3_. The positive effect of 0.5% CaCl_2_ on RWC and plant temperature can be attributed to the application of calcium prior to heat stress. While the direct measurement of malondialdehyde levels was not conducted in the study, it is speculated, based on information from the cited literature, that the application of calcium might have increased malondialdehyde levels. Elevated malondialdehyde stimulates the activity of key enzymes, including guaiacol peroxidase, superoxide dismutase (SOD), and catalase (CAT). These enzymes play pivotal roles in mitigating oxidative stress caused by heat. Guaiacol peroxidase scavenges reactive oxygen species (ROS), while SOD, and catalase decomposes hydrogen peroxide. By efficiently neutralizing oxidative stress, these enzymes collectively contribute to the plant’s enhanced heat tolerance, providing a mechanistic basis for the observed positive effects of CaCl_2_ application^47^. Further, the reduction in plant temperature resulting from the application of CaCl_2_ may be directly linked to its role in regulating chlorophyll content. Lower canopy temperatures reduce water loss through transpiration, thereby improving the water content of the plants. As a result, the application of 0.5% CaCl_2_ led to an improvement in the relative water content of cowpea and baby corn. These results confirm the findings of Palta^48^, Youssef et al*.*^46^, and Mubarik et al*.*^49^.

### Interaction

The 2:1 row ratio of cowpea + baby corn, combined with level of fertilizer at 150% RDF and the foliar application of CaCl_2_ at 0.5% during both the flowering and pod development stages of cowpea, resulted in marginally higher performance in nearly all parameters for both crops. This combination of factors appeared to create a mutually beneficial synergy, minimizing resource competition and optimizing resource utilization^30^. In the 2:1 row ratio, where baby corn plants were densely planted in close proximity to each other, they provided a shaded environment for cowpea, reducing temperature and transpiration from the surface and ultimately benefiting both crops^50^. Furthermore, different root systems and growth habits between cowpea and baby corn seemed to enable efficient resource utilization without excessive competition, enhancing overall resource use efficiency and yielding better growth^3^. The level of fertilizer at 150% RDF ensured an optimal nutrient supply for both crops within the 2:1 row ratio, guaranteeing essential resources for healthy growth and increased yields. Additionally, the foliar spray of CaCl_2_ at 0.5% during critical cowpea development stages played a pivotal role in stress mitigation by lowering plant temperature and increasing relative water content. Calcium chloride’s properties can reduce physiological stress, strengthen cell walls, and enhance nutrient uptake^45,46^. This specific combination of factors likely created a microenvironment in the plot that favoured the growth of both cowpea and baby corn. This microenvironment improvement could encompass better conditions for light penetration, air circulation, and soil quality. Furthermore, these factors might have had a positive, synergistic effect on the physiological processes of both crops, potentially enhancing the stress mitigation provided by the CaCl_2_ spray through improved nutrient supply from the chosen level of fertilizer. While, higher seed of cowpea and cob yield of baby corn during summer season were obtained in their respective sole crops fertilized with 150% RDF and spraying of CaCl_2_ 0.5% at flowering and pod development stage of cowpea over other combination. Yield decreased significantly in intercropping because of reduction in plant population. Devi and Singh^42^, Shukla et al.^51^ and Mndezebele et al.^52^ also reported the similar results.

## Conclusion

1. In conclusion, our study demonstrates that cultivating cowpea and baby corn in a 2:1 row ratio leads to significantly higher crop growth rate, relative growth rate, chlorophyll content, and relative water content for both crops (cowpea and baby corn). This row ratio also contributes to reducing canopy temperatures. Furthermore, the cowpea equivalent yield data indicate that this 2:1 row ratio is the more profitable choice during the summer season.
2. The results of fertilizer levels also showed that all the above-mentioned parameters (CGR, RGR and Chlorophyll content) of both crops were significantly higher with the fertilizer levels of 150% RDF (N_30_P_60_).
3. Among stress mitigating chemicals, foliar application of 0.5% CaCl_2_ had an overall positive effect on CGR, RGR, RWC of cowpea and baby corn as well as cowpea equivalent yield. Plant temperature cowpea and baby corn was significantly lowest with 0.5% CaCl_2_ as compare to KNO_3_ 1.0%. Hence, application of CaCl_2_ 0.5% during summer season is a viable option for reducing the adverse effect of higher temperature on cowpea and baby corn in the summer season. Generally, in summer season the stress mitigation in crops require more detailed research on how to improve the crop yield and how to minimize the higher temperature stress.
4. In summer season, intercropping of cowpea + baby corn in 2:1 row ratio with the fertilizer levels of 150% RDF (N_30_ P_60_) and foliar application of 0.5% CaCl_2_ had an overall positive effect on almost all the tested parameters of cowpea as well baby corn. Cowpea equivalent was also significantly improved by above mention treatment combination.

### Supplementary Information

Supplementary Tables.

## Data Availability

The datasets are not publicly available but may be obtained from the corresponding author upon reasonable request and with the permission of the institutions mentioned above.

## Code availability

The code may be obtained from the authors upon reasonable request.
