# Supplementary material for: Alleviating summer heat stress in cowpea-baby corn intercropping with stress-reducing chemicals and fertility variations
Source: Sci Rep. 2024 Feb 6;14:3020. doi: 10.1038/s41598-024-52862-2 (PMC10847103; doi:10.1038/s41598-024-52862-2)
Supplement: Supplementary file 1 — Supplementary Tables. [file 41598_2024_52862_MOESM1_ESM.docx]

**Supplementary table 1. *ANOVA* Table of cowpea - Dependent Variable is CGR at 25-40 & 40-55 DAS**

| **Source** | **At 25-40 DAS** | | | | | **At 40-55 DAS** | | | | |
| --- | --- | --- | --- | --- | --- | --- | --- | --- | --- | --- |
|  | **DF** | **Sum of Squares** | **Mean Square** | **F-Ratio** | **Significant** | **DF** | **Sum of Squares** | **Mean Square** | **F-Ratio** | **Significant** |
| Replication | 3 | 1.706 | 0.569 | 1.029 | NS | 3 | 0.444 | 0.148 | 0.218 | NS |
| C | 3 | 87.587 | 29.196 | 52.846 | * | 3 | 124.199 | 41.310 | 61.057 | * |
| Error(a) | 9 | 4.972 | 0.553 | . |  | 9 | 6.102 | 0.678 | . |  |
| F | 2 | 79.285 | 39.643 | 81.025 | * | 2 | 57.644 | 28.822 | 34.324 | * |
| C*F | 6 | 1.833 | 0.306 | 0.624 | NS | 6 | 5.963 | 0.994 | 1.184 | NS |
| Error(b) | 24 | 11.742 | 0.489 | . |  | 24 | 20.153 | 0.840 | . |  |
| S | 1 | 50.918 | 50.918 | 183.436 | * | 1 | 21.236 | 21.236 | 36.678 | * |
| C*S | 3 | 0.096 | 0.032 | 0.115 | NS | 3 | 2.322 | 0.774 | 1.337 | NS |
| F*S | 2 | 4.350 | 2.175 | 7.836 | * | 2 | 2.694 | 1.347 | 2.327 | NS |
| C*F*S | 6 | 1.582 | 0.264 | 0.950 | NS | 6 | 6.045 | 1.008 | 1.740 | NS |
| Error(c) | 36 | 9.993 | 0.278 | . |  | 36 | 20.843 | 0.579 | . |  |
| Total | 95 | 254.064 | . | . |  | 95 | 267.646 | . | . |  |

**Supplementary table 2. *ANOVA* Table of cowpea - Dependent Variable is RGR at 25-40 & 40-55 DAS**

| **Source** | **At 25-40 DAS** | | | | | **At 40-55 DAS** | | | | |
| --- | --- | --- | --- | --- | --- | --- | --- | --- | --- | --- |
|  | **DF** | **Sum of Squares** | **Mean Square** | **F-Ratio** | **Significant** | **DF** | **Sum of Squares** | **Mean Square** | **F-Ratio** | **Significant** |
| Replication | 3 | 0.4710 | 0.1570 | 0.7097 | NS | 3 | 0.2829 | 0.0943 | 0.5116 | NS |
| C | 3 | 4.4692 | 1.4897 | 6.7340 | * | 3 | 2.5466 | 0.8489 | 4.6044 | * |
| Error(a) | 9 | 1.9911 | 0.2212 | . |  | 9 | 1.6593 | 0.1844 | . |  |
| F | 2 | 5.0213 | 2.5107 | 8.3311 | * | 2 | 0.2982 | 0.1491 | 0.9403 | NS |
| C*F | 6 | 1.0175 | 0.1696 | 0.5627 | NS | 6 | 0.5781 | 0.0963 | 0.6076 | NS |
| Error(b) | 24 | 7.2327 | 0.3014 | . |  | 24 | 3.8055 | 0.1586 | . |  |
| S | 1 | 13.2021 | 13.2021 | 93.9589 | * | 1 | 0.1471 | 0.1471 | 1.2672 | NS |
| C*S | 3 | 0.0965 | 0.0322 | 0.2288 | NS | 3 | 0.5276 | 0.1759 | 1.5150 | NS |
| F*S | 2 | 0.7621 | 0.3810 | 2.7119 | NS | 2 | 0.6602 | 0.3301 | 2.8438 | NS |
| C*F*S | 6 | 0.7040 | 0.1173 | 0.8350 | NS | 6 | 0.9284 | 0.1547 | 1.3329 | NS |
| Error(c) | 36 | 5.0583 | 0.1405 | . |  | 36 | 4.1790 | 0.1161 | . |  |
| Total | 95 | 40.0257 | . | . |  | 95 | 15.6128 | . | . |  |

**Supplementary table 3. *ANOVA* Table of cowpea - Dependent Variable is chlorophyll content at 25 & 50 DAS**

| **Source** | **At 25 DAS** | | | | | **At 50 DAS** | | | | |
| --- | --- | --- | --- | --- | --- | --- | --- | --- | --- | --- |
|  | **DF** | **Sum of Squares** | **Mean Square** | **F-Ratio** | **Significant** | **DF** | **Sum of Squares** | **Mean Square** | **F-Ratio** | **Significant** |
| Replication | 3 | 0.0269 | 0.0090 | 1.9758 | NS | 3 | 0.0089 | 0.0030 | 3.003 | NS |
| C | 3 | 7.9937 | 2.6646 | 586.871 | * | 3 | 11.4767 | 3.8256 | 3866.745 | * |
| Error(a) | 9 | 0.0409 | 0.0045 | . |  | 9 | 0.0089 | 0.0010 | . |  |
| F | 2 | 0.2233 | 0.1117 | 14.736 | * | 2 | 0.2004 | 0.1002 | 17.723 | * |
| C*F | 6 | 0.0224 | 0.0037 | 0.493 | NS | 6 | 0.0053 | 0.0009 | 0.1563 | NS |
| Error(b) | 24 | 0.1818 | 0.0076 | . |  | 24 | 0.1357 | 0.0057 | . |  |
| S | 1 | 0.0060 | 0.0060 | 1.104 | NS | 1 | 0.1441 | 0.1441 | 18.991 | * |
| C*S | 3 | 0.0056 | 0.0019 | 0.341 | NS | 3 | 0.0038 | 0.0013 | 0.168 | NS |
| F*S | 2 | 0.0031 | 0.0016 | 0.285 | NS | 2 | 0.0012 | 0.0006 | 0.081 | NS |
| C*F*S | 6 | 0.0063 | 0.0010 | 0.192 | NS | 6 | 0.0190 | 0.0032 | 0.418 | NS |
| Error(c) | 36 | 0.1962 | 0.0055 | . |  | 36 | 0.2733 | 0.0076 | . |  |

**Supplementary table 4. *ANOVA* Table of cowpea - Dependent Variable is plant temperature at 25 & 50 DAS**

| **Source** | **At 25 DAS** | | | | | **At 50 DAS** | | | | |
| --- | --- | --- | --- | --- | --- | --- | --- | --- | --- | --- |
|  | **DF** | **Sum of Squares** | **Mean Square** | **F-Ratio** | **Significant** | **DF** | **Sum of Squares** | **Mean Square** | **F-Ratio** | **Significant** |
| Replication | 3 | 21.692 | 7.231 | 2.001 | NS | 3 | 47.277 | 15.759 | 8.439 | * |
| C | 3 | 8.089 | 2.696 | 0.746 | NS | 3 | 499.191 | 166.397 | 89.102 | * |
| Error(a) | 9 | 32.517 | 3.613 | . |  | 9 | 16.808 | 1.868 | . |  |
| F | 2 | 19.745 | 9.875 | 1.705 | NS | 2 | 5.585 | 2.792 | 0.377 | NS |
| C*F | 6 | 22.634 | 3.772 | 0.651 | NS | 6 | 51.474 | 8.579 | 1.157 | NS |
| Error(b) | 24 | 138.951 | 5.790 | . |  | 24 | 178.009 | 7.4170 | . |  |
| S | 1 | 12.760 | 12.760 | 1.781 | NS | 1 | 171.775 | 171.775 | 20.006 | * |
| C*S | 3 | 4.357 | 1.452 | 0.203 | NS | 3 | 1.213 | 0.404 | 0.047 | NS |
| F*S | 2 | 15.057 | 7.528 | 1.051 | NS | 2 | 8.3247 | 4.162 | 0.485 | NS |
| C*F*S | 6 | 39.211 | 6.535 | 0.912 | NS | 6 | 9.444 | 1.574 | 0.183 | NS |
| Error(c) | 36 | 257.865 | 7.162 | . |  | 36 | 309.111 | 8.586 | . |  |
| Total | 95 | 572.878 | . | . |  | 95 | 1298.211 | . | . |  |

**Supplementary table 5. *ANOVA* Table of cowpea - Dependent Variable is relative water content at 25 & 50 DAS**

| **Source** | **At 25 DAS** | | | | | **At 50 DAS** | | | | |
| --- | --- | --- | --- | --- | --- | --- | --- | --- | --- | --- |
|  | **DF** | **Sum of Squares** | **Mean Square** | **F-Ratio** | **Significant** | **DF** | **Sum of Squares** | **Mean Square** | **F-Ratio** | **Significant** |
| Replication | 3 | 52.593 | 17.531 | 0.987 | NS | 3 | 40.377 | 13.459 | 1.290 | NS |
| C | 3 | 52.497 | 17.499 | 0.985 | NS | 3 | 429.572 | 143.191 | 13.721 | * |
| Error(a) | 9 | 159.862 | 17.762 | . |  | 9 | 93.923 | 10.436 | . |  |
| F | 2 | 21.754 | 10.877 | 0.611 | NS | 2 | 46.275 | 23.137 | 1.637 | NS |
| C*F | 6 | 8.751 | 1.458 | 0.082 | NS | 6 | 9.757 | 1.626 | 0.115 | NS |
| Error(b) | 24 | 426.698 | 17.779 | . |  | 24 | 339.302 | 14.138 | . |  |
| S | 1 | 55.434 | 55.434 | 1.907 | NS | 1 | 244.641 | 244.641 | 11.589 | * |
| C*S | 3 | 19.118 | 6.373 | 0.219 | NS | 3 | 20.070 | 6.690 | 0.317 | NS |
| F*S | 2 | 1.025 | 0.513 | 0.018 | NS | 2 | 14.697 | 7.348 | 0.348 | NS |
| C*F*S | 6 | 2.280 | 0.380 | 0.013 | NS | 6 | 36.460 | 6.077 | 0.288 | NS |
| Error(c) | 36 | 1046.471 | 29.069 | . |  | 36 | 759.977 | 21.111 | . |  |
| Total | 95 | 1846.484 | . | . |  | 95 | 2035.048 | . | . |  |

- Significant at 5% (level of significance opted by user), NS - Non Significant, C=Intercropping system, F=Fertility level, S=Stress mitigating chemicals
p-Value < 0.05 - Significant at 5%, p-Value < 0.01 - Significant at 1%

**Supplementary table 6. *ANOVA* Table of baby corn - Dependent Variable is CGR at 25-40 & 40-55DAS**

| **Source** | **At 25-40 DAS** | | | | | **At 40-55 DAS** | | | | |
| --- | --- | --- | --- | --- | --- | --- | --- | --- | --- | --- |
|  | **DF** | **Sum of Squares** | **Mean Square** | **F-Ratio** | **Significant** | **DF** | **Sum of Squares** | **Mean Square** | **F-Ratio** | **Significant** |
| Replication | 3 | 4.705 | 1.568 | 1.728 | NS | 3 | 12.007 | 4.003 | 0.806 | NS |
| C | 3 | 1979.144 | 659.715 | 727.030 | * | 3 | 1453.737 | 484.579 | 97.606 | * |
| Error(a) | 9 | 8.167 | 0.907 | . |  | 9 | 44.682 | 4.965 | . |  |
| F | 2 | 166.248 | 83.124 | 36.931 | * | 2 | 389.445 | 194.722 | 82.714 | * |
| C*F | 6 | 9.189 | 1.532 | 0.680 | NS | 6 | 47.887 | 7.981 | 3.390 | * |
| Error(b) | 24 | 54.018 | 2.251 | . |  | 24 | 56.500 | 2.354 | . |  |
| S | 1 | 64.317 | 64.317 | 53.504 | * | 1 | 74.637 | 74.637 | 26.562 | * |
| C*S | 3 | 10.351 | 3.450 | 2.870 | * | 3 | 7.692 | 2.564 | 0.912 | NS |
| F*S | 2 | 2.731 | 1.365 | 1.136 | NS | 2 | 3.862 | 1.931 | 0.687 | NS |
| C*F*S | 6 | 5.932 | 0.989 | 0.822 | NS | 6 | 11.754 | 1.959 | 0.697 | NS |
| Error(c) | 36 | 43.276 | 1.202 | . |  | 36 | 101.159 | 2.810 | . |  |
| Total | 95 | 2348.076 | . | . |  | 95 | 2203.360 | . | . |  |

**Supplementary table 7. *ANOVA* Table of baby corn - Dependent Variable is RGR at 25-40 & 40-55 DAS**

| **Source** | **At 25-40 DAS** | | | | | | **At 40-55 DAS** | | | | |
| --- | --- | --- | --- | --- | --- | --- | --- | --- | --- | --- | --- |
|  | **DF** | **Sum of Squares** | **Mean Square** | **F-Ratio** | **Significant** | **DF** | | **Sum of Squares** | **Mean Square** | **F-Ratio** | **Significant** |
| Replication | 3 | 3.4157 | 1.1386 | 2.5100 | NS | 3 | | 1.3021 | 0.4340 | 1.1721 | NS |
| C | 3 | 4.8792 | 1.6264 | 3.5854 | NS | 3 | | 1.5682 | 0.5227 | 1.4116 | NS |
| Error(a) | 9 | 4.0826 | 0.4536 | . |  | 9 | | 3.3328 | 0.3703 | . |  |
| F | 2 | 0.2807 | 0.1403 | 0.1779 | NS | 2 | | 4.6613 | 2.3307 | 10.4298 | * |
| C*F | 6 | 0.8403 | 0.1401 | 0.1776 | NS | 6 | | 2.4844 | 0.4141 | 1.8530 | NS |
| Error(b) | 24 | 18.9274 | 0.7886 | . |  | 24 | | 5.3631 | 0.2235 | . |  |
| S | 1 | 6.2549 | 6.2549 | 11.9064 | * | 1 | | 0.9552 | 0.9552 | 5.1307 | * |
| C*S | 3 | 0.6340 | 0.2113 | 0.4023 | NS | 3 | | 2.0756 | 0.6919 | 3.7162 | * |
| F*S | 2 | 0.2691 | 0.1346 | 0.2561 | NS | 2 | | 0.0004 | 0.0002 | 0.0010 | NS |
| C*F*S | 6 | 1.9997 | 0.3333 | 0.6344 | NS | 6 | | 0.6086 | 0.1014 | 0.5448 | NS |
| Error(c) | 36 | 18.9124 | 0.5253 | . |  | 36 | | 6.7024 | 0.1862 | . |  |
| Total | 95 | 60.4960 | . | . |  | 95 | | 29.0541 | . | . |  |

**Supplementary table 8. *ANOVA* Table of baby corn - Dependent Variable is chlorophyll content at 25 & 50 DAS**

| **Source** | **At 25 DAS** | | | | | **At 50 DAS** | | | | |
| --- | --- | --- | --- | --- | --- | --- | --- | --- | --- | --- |
|  | **DF** | **Sum of Squares** | **Mean Square** | **F-Ratio** | **Significant** | **DF** | **Sum of Squares** | **Mean Square** | **F-Ratio** | **Significant** |
| Replication | 3 | 0.0055 | 0.0018 | 0.1997 | NS | 3 | 0.0165 | 0.0055 | 2.1171 | NS |
| C | 3 | 7.1526 | 2.3842 | 259.2258 | * | 3 | 12.0767 | 4.0256 | 1547.6688 | * |
| Error(a) | 9 | 0.0828 | 0.0092 | . |  | 9 | 0.0234 | 0.0026 | . |  |
| F | 2 | 0.1198 | 0.0599 | 17.3381 | * | 2 | 0.1364 | 0.0682 | 15.6402 | * |
| C*F | 6 | 0.0058 | 0.0010 | 0.2810 | NS | 6 | 0.0684 | 0.0114 | 2.6130 | * |
| Error(b) | 24 | 0.0829 | 0.0035 | . |  | 24 | 0.1047 | 0.0044 | . |  |
| S | 1 | 0.0004 | 0.0004 | 0.1209 | NS | 1 | 0.0213 | 0.0213 | 4.4574 | * |
| C*S | 3 | 0.0130 | 0.0043 | 1.3969 | NS | 3 | 0.1409 | 0.0470 | 9.8295 | * |
| F*S | 2 | 0.0072 | 0.0036 | 1.1576 | NS | 2 | 0.0003 | 0.0001 | 0.0277 | NS |
| C*F*S | 6 | 0.0039 | 0.0007 | 0.2115 | NS | 6 | 0.0224 | 0.0037 | 0.7822 | NS |
| Error(c) | 36 | 0.1120 | 0.0031 | . |  | 36 | 0.1720 | 0.0048 | . |  |
| Total | 95 | 7.5859 | . | . |  | 95 | 12.7830 | . | . |  |

**Supplementary table 9. *ANOVA* Table of baby corn - Dependent Variable is plant temperature at 25 & 50 DAS**

| **Source** | **At 25 DAS** | | | | | **At 50 DAS** | | | | |
| --- | --- | --- | --- | --- | --- | --- | --- | --- | --- | --- |
|  | **DF** | **Sum of Squares** | **Mean Square** | **F-Ratio** | **Significant** | **DF** | **Sum of Squares** | **Mean Square** | **F-Ratio** | **Significant** |
| Replication | 3 | 24.180 | 8.060 | 2.679 | NS | 3 | 73.644 | 24.548 | 2.571 | NS |
| C | 3 | 7.517 | 2.506 | 0.833 | NS | 3 | 455.856 | 151.952 | 15.914 | * |
| Error(a) | 9 | 27.082 | 3.009 | . |  | 9 | 85.934 | 9.548 | . |  |
| F | 2 | 0.030 | 0.015 | 0.002 | NS | 2 | 6.311 | 3.156 | 0.455 | NS |
| C*F | 6 | 23.90 | 3.984 | 0.490 | NS | 6 | 36.009 | 6.002 | 0.866 | NS |
| Error(b) | 24 | 195.049 | 8.127 | . |  | 24 | 166.300 | 6.929 | . |  |
| S | 1 | 12.028 | 12.028 | 1.644 | NS | 1 | 122.967 | 122.967 | 13.773 | * |
| C*S | 3 | 2.484 | 0.828 | 0.113 | NS | 3 | 8.122 | 2.707 | 0.303 | NS |
| F*S | 2 | 18.159 | 9.079 | 1.241 | NS | 2 | 4.246 | 2.123 | 0.238 | NS |
| C*F*S | 6 | 35.979 | 5.997 | 0.820 | NS | 6 | 24.067 | 4.011 | 0.449 | NS |
| Error(c) | 36 | 263.351 | 7.315 | . |  | 36 | 321.415 | 8.928 | . |  |
| Total | 95 | 609.761 | . | . |  | 95 | 1304.869 | . | . |  |

**Supplementary table 10. *ANOVA* Table of baby corn - Dependent Variable is relative water content at 25 & 50 DAS**

| **Source** | **At 25 DAS** | | | | | **At 50 DAS** | | | | |
| --- | --- | --- | --- | --- | --- | --- | --- | --- | --- | --- |
|  | **DF** | **Sum of Squares** | **Mean Square** | **F-Ratio** | **Significant** | **DF** | **Sum of Squares** | **Mean Square** | **F-Ratio** | **Significant** |
| Replication | 3 | 30.186 | 10.062 | 0.405 | NS | 3 | 88.937 | 29.645 | 2.647 | NS |
| C | 3 | 33.402 | 11.134 | 0.448 | NS | 3 | 23.473 | 7.824 | 0.699 | NS |
| Error(a) | 9 | 223.697 | 24.855 | . |  | 9 | 100.796 | 11.100 | . |  |
| F | 2 | 33.049 | 16.525 | 1.146 | NS | 2 | 17.490 | 8.745 | 0.465 | NS |
| C*F | 6 | 55.398 | 9.233 | 0.640 | NS | 6 | 19.878 | 3.313 | 0.176 | NS |
| Error(b) | 24 | 346.130 | 14.422 | . |  | 24 | 451.544 | 18.814 | . |  |
| S | 1 | 43.001 | 43.001 | 1.602 | NS | 1 | 255.552 | 255.552 | 15.780 | * |
| C*S | 3 | 49.228 | 16.409 | 0.611 | NS | 3 | 14.294 | 4.765 | 0.294 | NS |
| F*S | 2 | 33.168 | 16.584 | 0.618 | NS | 2 | 13.854 | 6.927 | 0.428 | NS |
| C*F*S | 6 | 14.351 | 2.392 | 0.089 | NS | 6 | 33.847 | 5.641 | 0.348 | NS |
| Error(c) | 36 | 966.147 | 26.837 | . |  | 3 | 88.937 | 29.646 | 2.647 | NS |
| Total | 95 | 1827.756 | . | . |  | 3 | 23.473 | 7.824 | 0.699 | NS |

- Significant at 5% (level of significance opted by user), NS - Non Significant, C=Intercropping system, F=Fertility level, S=Stress mitigating chemicals
p-Value < 0.05 - Significant at 5%, p-Value < 0.01 - Significant at 1%

**Supplementary table 11. Interactive effect of intercropping, fertility levels and stress mitigating chemicals on different parameters of cowpea**

|  | **MP_1_** | | | | **MP_2_** | | | | **MP_3_** | | | | **MP_4_** | | | |  |
| --- | --- | --- | --- | --- | --- | --- | --- | --- | --- | --- | --- | --- | --- | --- | --- | --- | --- |
| **MP_SP_SSP** | **SP_1_** | **SP_2_** | **SP_3_** | **Mean** | **SP_1_** | **SP_2_** | **SP_3_** | **Mean** | **SP_1_** | **SP_2_** | **SP_3_** | **Mean** | **SP_1_** | **SP_2_** | **SP_3_** | **Mean** | **Sub-Sub Plot Mean** |
| **Crop growth rate of cowpea at 25-40 DAS (g/m^2^/day)** | | | | | | | | | | | | | | | | | |
| SSP_1_ | 7.17(1.96) | 8.35(0.38) | 9.26(0.73) | 8.26(0.85) | 9.67(0.32) | 10.7(0.95) | 12.06(0.57) | 10.81(0.97) | 8.59(0.51) | 9.47(1.64) | 10.83(0.96) | 9.63(0.92) | 7.77(1.49) | 7.97(0.95) | 10.26(1.08) | 8.66(1.12) | 9.34(0.98) |
| SSP_2_ | 5.57(0.98) | 7.2(1.55) | 7.74(0.5) | 6.84(0.92) | 7.66(0.39) | 9.72(0.43) | 10.4(0.74) | 9.26(1.16) | 7.05(0.77) | 8.5(0.31) | 8.94(0.86) | 8.16(0.80) | 6.23(0.74) | 7.62(0.71) | 7.99(0.62) | 7.28(0.75) | 7.88(0.92) |
| Mean | 6.37(0.8) | 7.77(0.57) | 8.5(0.76) | 7.55(0.88) | 8.66(1.00) | 10.21(0.49) | 11.23(0.83) | B 10.03(0.77) | 7.82(0.77) | 8.99(0.48) | 9.89(0.94) | 8.9(0.73) | 7.0(0.77) | 7.79(0.17) | 9.13(1.13) | 7.97(0.87) | **8.61(0.95)** |
| **Crop growth rate of cowpea at 40-55 DAS (g/m2/day)** | | | | | | | | | | | | | | | | | |
| SSP_1_ | 4.92(0.16) | 6.22(0.08) | 7.45(0.19) | 6.2(1.26) | 6.92(0.15) | 9.05(0.04) | 10.72(0.04) | 8.9(1.90) | 6.61(0.06) | 7.5(0.05) | 7.72(0.04) | 7.28(0.58) | 5.86(0.09) | 7.05(0.1) | 7.11(0.06) | 6.68(0.70) | 7.26(1.01) |
| SSP_2_ | 4.21(0.11) | 4.87(0.09) | 5.78(0.12) | 4.95(0.78) | 7.45(0.1) | 8.11(0.05) | 9.05(0.05) | 8.21(0.84) | 6.39(0.06) | 6.28(0.06) | 7.44(0.04) | 6.7(0.64) | 4.3(0.08) | 5.42(0.07) | 6.55(0.06) | 5.42(1.12) | 6.32(1.27) |
| Mean | 4.56(0.50) | 5.54(0.95) | 6.61(1.80) | 5.57(0.88) | 7.19(0.37) | 8.58(0.66) | 9.89(1.18) | 8.55(0.48) | 6.5(0.15) | 6.89(0.86) | 7.58(0.19) | 6.99(0.41) | 5.08(1.10) | 6.24(1.15) | 6.83(0.39) | 6.05(0.89) | **6.79(0.66)** |
| **Relative growth rate of cowpea at 25-40 DAS (10^-2^ g/g/day)** | | | | | | | | | | | | | | | | | |
| SSP_1_ | 6.44(1.17) | 7.15(0.76) | 7.27(0.75) | 6.95(0.44) | 7.26(0.66) | 7.38(0.21) | 7.71(0.29) | 7.45(0.23) | 7.17(0.53) | 7.09(0.61) | 7.54(0.15) | 7.27(0.24) | 6.94(0.62) | 6.63(0.6) | 7.49(0.27) | 7.02(0.43) | 7.17(0.20) |
| SSP_2_ | 5.65(0.31) | 6.26 (0.87) | 6.43(0.72) | 6.11(0.41) | 6.28(0.16) | 6.92(0.1) | 7.08(0.31) | 6.76(0.42) | 6.32(0.32) | 6.6(0.39) | 6.61(0.3) | 6.51(0.16) | 6.05(0.19) | 6.51(0.36) | 6.46(0.43) | 6.34(0.25) | 6.43(0.23) |
| Mean | 6.05(0.55) | 6.70(0.62) | 6.85(0.59) | 6.53(0.59) | 6.77(0.69) | 7.15(0.32) | 7.40(0.44) | 7.11(0.48) | 6.74(0.60) | 6.85(0.34) | 7.07(0.65) | 6.89(0.57) | 6.50(0.69) | 6.57(0.08) | 6.98(0.72) | 6.68(0.48) | **6.80(0.52)** |
| **Relative growth rate of cowpea at 40-55 DAS (10^-2^ g/g/day)** | | | | | | | | | | | | | | | | | |
| SSP_1_ | 2.43(1.07) | 2.66(0.15) | 2.85(0.23) | 2.65(0.21) | 2.59(0.1) | 2.99(0.16) | 3.17(0.19) | 2.92(0.29) | 2.74(0.21) | 2.81(0.7) | 2.62(0.19) | 2.72(0.09) | 2.66(0.67) | 2.95(0.24) | 2.55(0.25) | 2.72(0.20) | 2.75(0.100) |
| SSP_2_ | 2.41(0.57) | 2.33(0.78) | 2.52(0.15) | 2.42(0.09) | 3.12(0.32) | 2.88(0.14) | 3.02(0.29) | 3.00(0.12) | 2.96(0.45) | 2.54(0.08) | 2.82(0.36) | 2.77(0.21) | 2.29(0.2) | 2.44(0.21) | 2.74(0.09) | 2.49(0.22) | 2.67(0.23) |
| Mean | 2.42(0.01) | 2.50(0.23) | 2.69(0.23) | 2.53(0.16) | 2.85(0.37) | 2.93(0.07) | 3.10(0.10) | 2.96(0.5) | 2.85(0.15) | 2.68(0.19) | 2.72(0.14) | 2.75(0.03) | 2.47(0.26) | 2.69(0.36) | 2.64(0.13) | 2.60(0.16) | **2.71(0.05)** |
| **Plant temperature of cowpea at 25 DAS (^0^C)** | | | | | | | | | | | | | | | | | |
| SSP_1_ | 27.88(3.07) | 25.63(2.1) | 27.38(2.17) | 26.96(1.18) | 24.28(2.15) | 26.53(3.91) | 28.3(3.35) | 26.37(2.01) | 25.88(2.95) | 28.38(2.62) | 27.38(4.17) | 27.21(1.26) | 25.18(2.58) | 27.53(2.62) | 28.43(2.47) | 27.04(1.67) | 26.89(0.31) |
| SSP_2_ | 25.75(2.63) | 27.25(2.02) | 27.13(2.2) | 26.71(0.83) | 25.23(1.77) | 25.35(2.7) | 26.75(3.41) | 25.78(0.84) | 26.13(2.3) | 25.88(2.96) | 25.38(2) | 25.79(0.38) | 27.18(1.07) | 26.43(2.59) | 25.55(1.85) | 26.38(0.82) | 26.16(0.40) |
| Mean | 26.81(1.50) | 26.44(1.14) | 27.25(0.17) | 26.83(0.17) | 24.75(0.67) | 25.94(0.83) | 27.53(1.09) | 26.07(0.42) | 26(0.17) | 27.13(1.76) | 26.38(1.41) | 26.5(1.00) | 26.18(1.41) | 26.98(0.77) | 26.99(2.03) | 26.71(0.46) | **26.53(0.52)** |
| **Plant temperature of cowpea at 50 DAS (^0^C)** | | | | | | | | | | | | | | | | | |
| SSP_1_ | 32.42(4.71) | 33.79(2.17) | 32.17(2.07) | 32.79(0.87) | 27.88(3.94) | 25.69(3.58) | 25.63(2.75) | 26.4(1.28) | 27.99(4.01) | 27.61(1.84) | 27.98(3.59) | 27.86(0.22) | 28.81(3.95) | 28.28(1.84) | 29.44(3.43) | 28.84(0.58) | 28.97(2.37) |
| SSP_2_ | 34.17(1.51) | 34.92(1.75) | 36.54(2.53) | 35.21(1.21) | 31.23(5.6) | 28.69(1.95) | 28.31(2.02) | 29.41(1.58) | 29.99(2.84) | 30.11(2.8) | 31.11(3.44) | 30.4(0.61) | 30.66(2.83) | 30.78(2.37) | 33.28(3.44) | 31.57(1.48) | 31.65(2.19) |
| Mean | 33.29(1.23) | 34.35(0.79) | 34.35(3.09) | 34(1.71) | 29.55(2.36) | 27.19(2.12) | 26.97(1.89) | 27.9(2.13) | 28.99(1.41) | 28.86(1.77) | 29.55(2.21) | 29.13(1.80) | 29.73(1.31) | 29.53(1.77) | 31.36(2.71) | 30.21(1.93) | **30.31(1.90)** |
| **Relative water content in cowpea at 25 DAS (%)** | | | | | | | | | | | | | | | | | |
| SSP_1_ | 70.13(5.08) | 70.13(6.49) | 70.25(6.25) | 70.17(0.07) | 70.38(5.58) | 71.38(4.31) | 71.75(2.82) | 71.17(0.71) | 69.5(4.32) | 70.63(7.21) | 71.75(6.79) | 70.63(1.13) | 68.21(4.19) | 68.99(5.39) | 69.61(2.28) | 68.94(0.70) | 70.22(0.82) |
| SSP_2_ | 67.75(4.65) | 66.88(1.96) | 68.25(3.81) | 67.63(0.69) | 69.38(6.05) | 70.13(5.28) | 70.88(3.52) | 70.13(0.75) | 67.56(2.91) | 68.31(2.88) | 69.44(2.01) | 68.44(0.95) | 68.46(8.83) | 68.71(2.59) | 68.71(2.44) | 68.63(0.14) | 68.7(0.90) |
| Mean | 68.94(1.68) | 68.5(2.29) | 69.25(1.41) | 68.9(1.80) | 69.88(0.71) | 70.75(0.88) | 71.31(0.62) | 70.65(0.74) | 68.53(1.37) | 69.47(1.64) | 70.59(1.63) | 69.53(1.55) | 68.34(0.18) | 68.85(0.20) | 69.16(0.64) | 68.78(0.22) | **69.46(1.07)** |
| **Relative water content in cowpea at 50 DAS (%)** | | | | | | | | | | | | | | | | | |
| SSP_1_ | 71.48(0.52) | 68.48(2.98) | 72.60(0.1) | 70.85(2.13) | 74.38().4) | 75.13(0.38) | 76.63(1.0) | 75.38(1.14) | 71.48(1.48) | 71.63(0.26) | 73.06(0.06) | 74.56(0.87) | 73.08(0.08) | 69.25(0.024) | 70.06(0.5) | 71.00(2.02) | 72.94(2.04) |
| SSP_2_ | 65.23(0.23) | 68.23(2.23) | 66.60(0.1) | 66.68(1.50) | 71.88(0.63) | 72.50(0.5) | 74.00(0.5) | 72.79(1.09) | 65.23(0.23) | 67.88(.01) | 69.50(0.1) | 69.88(2.16) | 69.08(0.08) | 68.25(68.25) | 67.88(0.049) | 68.12(0.62) | 68.08(2.28) |
| Mean | 68.35(4.12) | 68.35(0.18) | 69.60(4.24) | 68.77(2.95) | 73.13(1.77) | 73.81(1.86) | 75.31(1.86) | 74.08(1.83) | 68.35(4.42) | 69.75(2.65) | 71.28(2.52) | 72.22(3.31) | 71.08(2.83) | 68.75(0.71) | 68.97(1.54) | 69.56(2.04) | 69.09(1.43) |
| **Chlorophyll content in cowpea at 25 DAS (mg/g)** | | | | | | | | | | | | | | | | | |
| SSP_1_ | 1.66(1.78) | 1.70(0.85) | 1.75(0.72) | 1.70(0.05) | 2.4(0.69) | 2.47(0.58) | 2.52(0.7) | 2.46(0.06) | 2.07(0.82) | 2.12(1.4) | 2.22(0.66) | 2.14(0.08) | 1.76(1.21) | 1.89(0.75) | 1.92(0.55) | 1.85(0.09) | 2.04(0.29) |
| SSP_2_ | 1.63(0.84) | 1.67(1.47) | 1.73(0.63) | 1.67(0.05) | 2.41(0.63) | 2.43(0.56) | 2.49(0.92) | 2.44(0.04) | 2.08(0.75) | 2.11(0.55) | 2.16(0.82) | 2.11(0.04) | 1.77(0.62) | 1.88(0.73) | 1.94(0.74) | 1.87(0.086) | 2.02(0.29) |
| Mean | 1.64(0.02) | 1.69(0.02) | 1.74(0.01) | 1.69(0.02) | 2.40(0.007) | 2.45(0.03) | 2.50(0.02) | 2.45(0.01) | 2.08(0.007) | 2.11(0.007) | 2.19(0.04) | 2.12(0.02) | 1.77(0.007) | 1.88(0.007) | 1.93(0.01) | 1.86(0.014) | **2.03(0.01)** |
| **Chlorophyll content in cowpea at 50 DAS (mg/g)** | | | | | | | | | | | | | | | | | |
| SSP_1_ | 1.85(0.02) | 1.95(0.02) | 2(0.16) | 1.94(0.08) | 2.79(0.06) | 2.83(0.03) | 2.89(0.04) | 2.84(0.05) | 2.31(0.04) | 2.39(0.04) | 2.4(0.04) | 2.37(0.05) | 2.02(0.04) | 2.05(0.19) | 2.12(0.05) | 2.06(0.5) | 2.30(0.35) |
| SSP_2_ | 1.8(0.02) | 1.86(0.02) | 1.87(0.02) | 1.84(0.04) | 2.68(0.1) | 2.74(0.04) | 2.82(0.04) | 2.75(0.07) | 2.26(0.04) | 2.27(0.03) | 2.38(0.13) | 2.30(0.07) | 1.94(0.11) | 2.01(0.03) | 2.05(0.04) | 2.00(0.06) | 2.22(0.25) |
| Mean | 1.83(0.04) | 1.91(0.06) | 1.94(0.09) | 1.89(0.07) | 2.73(0.08) | 2.79(0.06) | 2.86(0.05) | 2.79(0.06) | 2.28(0.03) | 2.33(0.08) | 2.39(0.01) | 2.33(0.05) | 1.98(0.06) | 2.03(0.03) | 2.09(0.05) | 2.03(0.04) | **2.26(0.06)** |
| **Seed yield (kg/ha)** | | | | | | | | | | | | | | | | | |
| SSP_1_ | 730(68.21) | 774(19.60) | 810(12.17) | 771(32.71) | 456(42.92) | 534(16.58) | 600(71.53) | 530(58.86) | 487(39.37) | 592(41.29) | 631(25.50) | 570(60.81) | 581(7.04) | 635(24.77) | 650(19.83) | 622(29.63) | 623(91.33) |
| SSP_2_ | 698(71.78) | 739(40.47) | 785(34.93) | 741(35.54) | 470(21.94) | 495(24.59) | 523(45.69) | 496(22.05) | 484(7.63) | 535(28.61) | 579(35.62) | 533(38.82) | 539(28.46) | 580(29.30) | 650(37.07) | 590(45.82) | 590(93.39) |
| Mean | 714(16.00) | 757(17.5) | 798(12.5) | 756(33.89) | 463(6.5) | 515(19.5) | 561(38.5) | 513(40.03) | 485(1.5) | 564(28.5) | 605(26.0) | 551(49.88) | 560(21) | 608(27.5) | 650(0.0) | 606(36.75) | **606(92.43)** |

MP - Main Plot, SP - Sub Plot, SSP - Sub-Sub Plot MP_1_= Sole cowpea, MP_2_= Cowpea + Baby corn (2:1), MP_3_= Cowpea + Baby corn (3:1), MP_4_= Cowpea + Baby corn (4:1)

SP_1_=100 % RDF, SP_2_=125% RDF, SP_3_=150% RDF

SSP_1=_0.5% CaCl_2_ SSP_2=_1% KNO_3_

**Supplementary table 12. Critical Difference (CD) values at 5% for different parameters of cowpea (referring to table 12)**

| **Source** | **CGR 25-40** | **CGR 40-55** | **RGR 25-40** | **RGR 40-55** | **Plant Temp. 25 DAS** | **Plant Temp. 50 DAS** | **RWC 25 DAS** | **RWC 50 DAS** | **Chlorophyll content at 25 DAS** | **Chlorophyll content at 50 DAS** | **Grain yield** |
| --- | --- | --- | --- | --- | --- | --- | --- | --- | --- | --- | --- |
| Main plot - MP Means | 0.49 | 0.54 | 0.31 | 0.28 | NS | 0.89 | NS | 2.11 | 0.04 | 0.02 | 0.19 |
| Sub plot - SP Means | 0.36 | 0.47 | 0.28 | NS | NS | NS | NS | NS | 0.04 | 0.04 | 0.19 |
| Sub-Sub plot - SSP Means | 0.22 | 0.31 | 0.16 | NS | NS | 1.21 | NS | 1.9 | NS | 0.04 | 0.10 |
| SP Means at the same or different levels of MP | NS | NS | NS | NS | NS | NS | NS | NS | NS | NS | NS |
| MP Means at the same or different levels of SP | NS | NS | NS | NS | NS | NS | NS | NS | NS | NS | NS |
| SSP Means at the same level of MP | NS | NS | NS | NS | NS | NS | NS | NS | NS | NS | NS |
| MP Means at the same or different levels of SSP | NS | NS | NS | NS | NS | NS | NS | NS | NS | NS | NS |
| SSP Means at the same level of SP | 0.38 | NS | NS | NS | NS | NS | NS | NS | NS | NS | NS |
| SP Means at the same or different levels of SSP | 0.45 | NS | NS | NS | NS | NS | NS | NS | NS | NS | NS |
| SSP Means at the same combination of MP and SP | NS | NS | NS | NS | NS | NS | NS | NS | NS | NS | NS |
| SP Means at the same combination of MP and SSP | NS | NS | NS | NS | NS | NS | NS | NS | NS | NS | NS |
| MP Means at the same combination of SP and SSP | NS | NS | NS | NS | NS | NS | NS | NS | NS | NS | NS |
| NS - Non Significant, MP=Main plot, SP=Sub plot, SSP=Sub-sub plot | | | | | | | | | | |  |

**Supplementary table 13. Interactive effect of intercropping, fertility levels and stress mitigating chemicals on different parameters of baby corn**

|  | **MP_1_** | | | | **MP_2_** | | | | **MP_3_** | | | | **MP_4_** | | | |  |
| --- | --- | --- | --- | --- | --- | --- | --- | --- | --- | --- | --- | --- | --- | --- | --- | --- | --- |
| **MP_SP_SSP** | **SP_1_** | **SP_2_** | **SP_3_** | **Mean** | **SP_1_** | **SP_2_** | **SP_3_** | **Mean** | **SP_1_** | **SP_2_** | **SP_3_** | **Mean** | **SP_1_** | **SP_2_** | **SP_3_** | **Mean** | **Sub-Sub Plot Mean** |
| **Crop growth rate of baby corn at 25-40 DAS (g/m^2^/day)** | | | | | | | | | | | | | | | | | |
| SSP_1_ | 8.08(0.02) | 9.59(0.02) | 10.69(0.16) | 9.45(1.31) | 20.2(0.06) | 22.48(0.03) | 24.64(0.04) | 22.44(2.22) | 15.98(0.04) | 17.92(0.04) | 19.59(0.04) | 17.83(1.81) | 14.92(0.04) | 17.14(0.19) | 18.7(0.05) | 16.92(1.90) | 16.66(4.66) |
| SSP_2_ | 7.22(0.02) | 8.6(0.02) | 10.06(0.02) | 8.63(1.42) | 19.14(0.1) | 21.11(0.04) | 22.89(0.04) | 21.05(1.88) | 14.56(0.04) | 16(0.03) | 17.84(0.13) | 16.13(1.64) | 13.09(0.11) | 15.31(0.03) | 14.45(0.04) | 14.28(1.12) | 15.02(4.44) |
| Mean | 7.65(0.61) | 9.09(0.70) | 10.37(0.45) | 9.04(0.58) | 19.67(0.75) | 21.80(0.97) | 23.77(1.24) | 21.74(0.98) | 15.27(1.00) | 16.96(1.36) | 18.71(1.24) | 16.98(1.20) | 14.00(1.29) | 16.23(1.29) | 16.57(3.01) | 15.60(1.87) | **15.84(1.16)** |
| **Crop growth rate of baby corn at 40-55 DAS (g/m^2^/day)** | | | | | | | | | | | | | | | | | |
| SSP_1_ | 5.9(0.16) | 8.32(0.08) | 8.83(0.19) | 7.68(1.57) | 16.41(0.15) | 18.35(0.04) | 20.8(0.04) | 18.52(2.20) | 10.41(0.06) | 13.85(0.05) | 17.52(0.04) | 13.93(3.56) | 8.69(0.09) | 11.3(0.1) | 15.58(0.06) | 11.85(3.47) | 13.00(3.90) |
| SSP_2_ | 4.51(0.11) | 5.53(0.09) | 6.68(0.12) | 5.57(1.09) | 13.52(0.1) | 17.74(0.05) | 18.46(0.05) | 16.58(2.67) | 8.8(0.06) | 12.58(0.06) | 13.8(0.04) | 11.72(2.61) | 8.47(0.08) | 10.24(0.07) | 14.47(0.06 | 11.06(3.08) | 11.23(3.90) |
| Mean | 5.21(0.98) | 6.92(1.97) | 7.76(1.52) | 6.63(1.49) | 14.97(2.04) | 18.05(0.43) | 19.63(1.65) | 17.55(1.37) | 9.60(1.14) | 13.22(0.90) | 15.66(2.63) | 12.83(1.56) | 8.58(0.16) | 10.77(0.75) | 15.02(0.78) | 11.46(0.56) | **12.11(1.25)** |
| **Relative growth rate of baby corn at 25-40 DAS (10^-2^ g/g/day)** | | | | | | | | | | | | | | | | | |
| SSP_1_ | 11.08(1.17) | 11.22(0.76) | 11.07(0.75) | 11.13(0.08) | 11.42(0.66) | 11.42(0.21) | 11.53(0.29) | 11.45(0.06) | 10.96(0.53) | 11.05(0.61) | 11.41(0.15) | 11.14(0.24) | 10.9(0.62) | 11.03(0.6) | 11.38(0.27) | 11.10(0.24) | 11.21(0.14) |
| SSP_2_ | 10.37(0.31) | 10.45(0.87) | 10.82(0.72) | 10.55(0.24) | 11.26(0.16) | 11.13(0.1) | 11.27(0.31) | 11.22(0.08) | 10.46(0.32) | 10.49(0.39) | 10.73(0.3) | 10.56(0.15) | 10.62(0.19) | 10.8(0.36) | 9.93(0.43 | 10.45(0.46) | 10.70(0.31) |
| Mean | 10.72(0.50) | 10.84(0.54) | 10.95(0.18) | 10.84(0.41) | 11.34(0.11) | 11.28(0.21) | 11.40(0.18) | 11.34(0.16) | 10.71(0.35) | 10.77(0.40) | 11.07(0.48) | 10.85(0.41) | 10.76(0.19) | 10.92(0.16) | 10.65(1.03) | 10.78(0.46) | **10.95(0.36)** |
| **Relative growth rate of baby corn at 40-55 DAS (10^-2^ g/g/day)** | | | | | | | | | | | | | | | | | |
| SSP_1_ | 3.12(1.07) | 3.53(0.15) | 3.41(0.23) | 3.35(0.21) | 3.4(0.1) | 3.43(0.16) | 3.5(0.19) | 3.44(0.05) | 2.82(0.21) | 3.24(0.7) | 3.66(0.19) | 3.24(0.42) | 2.57(0.67) | 2.85(0.24) | 3.47(0.25) | 2.96(0.46) | 3.25(0.18) |
| SSP_2_ | 2.66(0.57) | 2.71(0.78) | 2.83(0.15) | 2.73(0.09) | 3.03(0.32) | 3.46(0.14) | 3.37(0.29) | 3.29(0.23) | 2.6(0.45) | 3.22(0.08) | 3.2(0.36) | 3.01(0.35) | 2.79(0.2) | 2.87(0.21) | 3.85(0.09 | 3.17(0.59) | 3.05(0.21) |
| Mean | 2.89(0.33) | 3.12(0.58) | 3.12(0.41) | 3.04(0.44) | 3.21(0.26) | 3.45(0.02) | 3.43(0.09) | 3.37(0.11) | 2.71(0.16) | 3.23(0.01) | 3.43(0.33) | 3.12(0.16) | 2.68(0.16) | 2.86(0.01) | 3.66(0.27) | 3.07(0.15) | **3.15(0.14)** |
| **Plant temperature of baby corn at 25 DAS (^0^C)** | | | | | | | | | | | | | | | | | |
| SSP_1_ | 27.88(3.07) | 25.23(2.1) | 26.38(2.17) | 26.49(1.33) | 24.38(2.15) | 26.65(3.91) | 28.25(3.35) | 26.43(1.94) | 26(2.95) | 27.7(2.62) | 26.55(4.17) | 26.75(0.87) | 26.38(2.58) | 27.34(2.62) | 27.75(2.47 | 27.16(0.70) | 26.71(0.29) |
| SSP_2_ | 26(2.63) | 26.89(2.02) | 26.13(2.2) | 26.34(0.48) | 25.83(1.77) | 24.9(2.7) | 25.73(3.41) | 25.48(0.51) | 26.6(2.3) | 25.87(2.96) | 25.1(2) | 25.86(0.75) | 27.63(1.07) | 26.44(2.59) | 24.88(1.85) | 26.32(1.38) | 26.00(0.36) |
| Mean | 26.94(1.33) | 26.06(1.17) | 26.25(1.18) | 26.41(0.11) | 25.10(1.03) | 25.78(1.24) | 26.99(1.78) | 25.95(0.67) | 26.30(0.42) | 26.79(1.29) | 25.82(1.03) | 26.30(0.63) | 27.01(0.88) | 26.89(0.64) | 26.32(2.03) | 26.74(0.59) | **26.35(0.28)** |
| **Plant temperature of baby corn at 50 DAS (^0^C)** | | | | | | | | | | | | | | | | | |
| SSP_1_ | 38.34(4.71) | 38.82(2.17) | 36.84(2.07) | 38.00(1.03) | 31.89(3.94) | 31.14(3.58) | 33.21(2.75) | 32.08(1.05) | 31.71(4.01) | 34.09(1.84) | 31.84(3.59) | 32.54(1.34) | 33.17(3.95) | 34.42(1.84) | 33.79(3.43 | 33.79(0.62) | 34.10(2.34) |
| SSP_2_ | 40.67(1.51) | 39.09(1.75) | 38.84(2.53) | 39.53(0.99) | 32.71(5.6) | 34.21(1.95) | 34.89(2.02) | 33.93(1.12) | 34.21(2.84) | 35.09(2.8) | 36.96(3.44) | 35.42(1.40) | 36.42(2.83) | 37.17(2.37) | 36.17(3.44) | 36.58(0.52) | 36.37(2.05) |
| Mean | 39.50(1.65) | 38.95(0.19) | 37.84(1.41) | 38.76(1.80) | 32.30(0.58) | 32.67(2.17) | 34.05(1.88) | 33.01(1.30) | 32.96(1.77) | 34.59(0.71) | 34.40(3.62) | 33.98(2.03) | 34.79(2.30) | 35.79(10.94) | 34.98(1.68) | 35.19(1.97) | **35.23(1.61)** |
| **Relative water content in baby corn at 25 DAS (%)** | | | | | | | | | | | | | | | | | |
| SSP_1_ | 73.38(5.08) | 75.75(6.49) | 76.35(6.25) | 75.16(1.57) | 75.05(5.58) | 74.49(4.31) | 75.06(2.82) | 74.87(0.33) | 72.38(4.32) | 75(7.21) | 78.5(6.79) | 75.29(3.07) | 72.93(4.19) | 74.55(5.39) | 75.05(2.28 | 74.18(1.11) | 74.87(0.45) |
| SSP_2_ | 72.06(4.65) | 70.25(1.96) | 72.14(3.81) | 71.48(1.07) | 76.3(6.05) | 73.94(5.28) | 74.2(3.52) | 74.81(1.29) | 73(2.91) | 74.25(2.88) | 74.63(2.01) | 73.96(0.85) | 73.43(8.83) | 74.3(2.59) | 73.93(2.44) | 73.88(0.44) | 73.53(1.24) |
| Mean | 72.72(0.93) | 73.00(3.89) | 74.24(2.98) | 73.32(2.60) | 75.68(0.88) | 74.21(0.39) | 74.63(0.61) | 74.84(0.04) | 72.69(0.44) | 74.63(0.53) | 76.56(2.74) | 74.63(0.94) | 73.18(0.35) | 74.43(0.18) | 74.49(0.79) | 74.03(0.21) | **74.20(0.95)** |
| **Relative water content in baby corn at 50 DAS (%)** | | | | | | | | | | | | | | | | | |
| SSP_1_ | 77.84(2.69) | 78.46(5.4) | 78.34(5.75) | 78.21(0.33) | 77.18(4.24) | 79.93(2.17) | 79.3(3.89) | 78.80(1.44) | 76.2(2.28) | 78.39(5.7) | 81.06(6.33) | 78.55(2.43) | 77.63(4.08) | 77.75(4.56) | 77.25(3.18 | 77.54(0.26) | 78.24(0.53) |
| SSP_2_ | 73.46(2.08) | 73.71(5.64) | 74.21(2.81) | 73.79(0.38) | 75.8(4.82) | 74.43(3.74) | 77.3(2.27) | 75.84(1.43) | 75.41(1.66) | 75.06(2.52) | 75.03(1.41) | 75.17(0.21) | 75.38(8.56) | 75.63(2.97) | 74.75(2.76) | 75.25(0.45) | 75.01(0.75) |
| Mean | 75.65(3.09) | 76.09(3.36) | 76.27(2.93) | 76.00(3.13) | 76.49(0.98) | 77.18(3.89) | 78.30(1.41) | 77.32(2.09) | 75.80(0.59) | 76.73(2.35) | 78.05(4.26) | 76.86(2.39) | 76.50(1.59) | 76.69(1.49) | 76.00(1.77) | 76.40(1.62) | **76.65(0.49)** |
| **Chlorophyll content in baby corn at 25 DAS (mg/g)** | | | | | | | | | | | | | | | | | |
| SSP_1_ | 1.12(1.78) | 1.17(0.85) | 1.18(0.72) | 1.16(0.03) | 1.86(0.69) | 1.96(0.58) | 1.97(0.7) | 1.93(0.06) | 1.54(0.82) | 1.6(1.4) | 1.65(0.66) | 1.60(0.06) | 1.35(1.21) | 1.43(0.75) | 1.44(0.55) | 1.41(0.05) | 1.52(0.28) |
| SSP_2_ | 1.17(0.84) | 1.17(1.47) | 1.24(0.63) | 1.19(0.04) | 1.87(0.63) | 1.91(0.56) | 1.95(0.92) | 1.91(0.04) | 1.55(0.75) | 1.58(0.55) | 1.61(0.82) | 1.58(0.03) | 1.35(0.62) | 1.39(0.73) | 1.45(0.74 | 1.39(0.05) | 1.52(0.27) |
| Mean | 1.15(0.04) | 1.17(0) | 1.21(0.04) | 1.17(0.02) | 1.87(0.007) | 1.93(0.04) | 1.96(0.01) | 1.92(0.01) | 1.54(0.007) | 1.59(0.01) | 1.63(0.03) | 1.59(0.01) | 1.35(0) | 1.41(0.03) | 1.44(0.007) | 1.40(0.01) | **1.52(0.27)** |
| **Chlorophyll content in baby corn at 50 DAS (mg/g)** | | | | | | | | | | | | | | | | | |
| SSP_1_ | 1.25(1.96) | 1.35(0.38) | 1.3(0.73) | 1.30(0.05) | 2.28(0.32) | 2.32(0.95) | 2.37(0.57) | 2.32(0.04) | 1.75(0.51) | 1.83(1.64) | 1.84(0.96) | 1.81(0.05) | 1.48(1.49) | 1.51(0.95) | 1.58(1.08) | 1.52(0.05) | 1.74(0.38) |
| SSP_2_ | 1.36(0.98) | 1.49(1.55) | 1.36(0.5) | 1.40(0.075) | 2.17(0.39) | 2.23(0.43) | 2.3(0.74) | 2.23(0.067) | 1.7(0.77) | 1.71(0.31) | 1.82(0.86) | 1.74(0.07) | 1.4(0.74) | 1.46(0.71) | 1.51(0.62 | 1.46(0.05) | 1.71(0.33) |
| Mean | 1.31(0.08) | 1.42(0.10) | 1.33(0.04) | 1.35(0.07) | 2.22(0.08) | 2.28(0.06) | 2.34(0.05) | 2.28(0.06) | 1.72(0.04) | 1.77(0.08) | 1.83(0.01) | 1.77(0.05) | 1.44(0.05) | 1.49(0.04) | 1.55(0.05) | 1.49(0.04) | **1.72(0.36)** |
| **Raw cob yield (kg/ha)** | | | | | | | | | | | | | | | | | |
| SSP_1_ | 4116 (87.27) | 4176(253.95) | 4329(194.75) | 4207(89.68) | 2128(112.26) | 2323(116.42) | 2418(48.01) | 2290(120.72) | 1616(183.5) | 1677(216.48) | 1768(170.88) | 1687(62.45) | 1013(67.73) | 1170(194.86) | 1276(87.28) | 1153(108.04) | 2334(1153.62) |
| SSP_2_ | 4029 (40.01) | 4056(53.30) | 4193(229.80) | 4093(71.80) | 2078(55.72) | 2241(199.57) | 2328(173.83) | 2216(103.62) | 1579(90.51) | 1662(179.48) | 1714(83.62) | 1652(55.60) | 938(146.67) | 1070(80.04) | 1124(227.99) | 1044(78.12) | 2251(1141.25) |
| Mean | 4073 (43.5) | 4116(60.00) | 4261(68.00) | 4150(80.43) | 2103 (25.0) | 2282(41.0) | 2373(45.00) | 2253(112.16) | 1598(18.5) | 1669(7.50) | 1741(27.0) | 1669(58.37) | 975(37.50) | 1120(50.0) | 1200(76.00) | 1098(93.12) | **2293(1147.54)** |

MP - Main Plot, SP - Sub Plot, SSP - Sub-Sub Plot MP_1_= Sole Baby corn, MP_2_= Cowpea + Baby corn (2:1), MP_3_= Cowpea + Baby corn (3:1), MP_4_= Cowpea + Baby corn (4:1), SP_1_=100 % RDF, SP_2_=125% RDF, SP_3_=150% RDF, SSP_1=_0.5% CaCl_2_ SSP_2=_1% KNO_3_

**Supplementary table 14. Critical Difference (CD) values at 5% for different parameters of baby corn (referring to table 12)**

| **Source** | **CGR 20-40 DAS** | **CGR 40-55 DAS** | **RGR 25-40 DAS** | **RGR 40-55 DAS** | **Plant Temp. 25 DAS** | **Plant Temp. 50 DAS** | **RWC 25 DAS** | **RWC 50 DAS** | **Chlorophyll content at 25 DAS** | **Chlorophyll content at 50 DAS** | **Cob yield** |
| --- | --- | --- | --- | --- | --- | --- | --- | --- | --- | --- | --- |
| Main plot - MP Means | 0.62 | 1.46 | NS | NS | NS | 2.02 | NS | NS | 0.06 | 0.03 | 97.90 |
| Sub plot - SP Means | 0.77 | 0.79 | NS | 0.24 | NS | NS | NS | NS | 0.03 | 0.03 | 90.53 |
| Sub-Sub plot - SSP Means | 0.45 | 0.69 | 0.30 | 0.18 | NS | 1.24 | NS | 1.67 | NS | 0.03 | 75.37 |
| SP Means at the same or different levels of MP | NS | 1.58 | NS | NS | NS | NS | NS | NS | NS | 0.07 | NS |
| MP Means at the same or different levels of SP | NS | 1.94 | NS | NS | NS | NS | NS | NS | NS | 0.06 | NS |
| SSP Means at the same level of MP | 0.91 | NS | NS | 0.36 | NS | NS | NS | NS | NS | 0.06 | NS |
| MP Means at the same or different levels of SSP | 0.89 | NS | NS | 0.47 | NS | NS | NS | NS | NS | 0.05 | NS |
| SSP Means at the same level of SP | NS | NS | NS | NS | NS | NS | NS | NS | NS | NS | NS |
| SP Means at the same or different levels of SSP | NS | NS | NS | NS | NS | NS | NS | NS | NS | NS | NS |
| SSP Means at the same combination of MP and SP | NS | NS | NS | NS | NS | NS | NS | NS | NS | NS | NS |
| SP Means at the same combination of MP and SSP | NS | NS | NS | NS | NS | NS | NS | NS | NS | NS | NS |
| MP Means at the same combination of SP and SSP | NS | NS | NS | NS | NS | NS | NS | NS | NS | NS | NS |
| NS - Non Significant, MP=Main plot, SP=Sub plot, SSP=Sub-sub plot | | | | | | | | | | |  |

**Supplementary table 15. Interactive effect of intercropping, fertility levels and stress mitigating chemicals on cowpea equivalent yield**

|  | **MP_1_** | | | | **MP_2_** | | | | **MP_3_** | | | | **MP_4_** | | | | **MP_5_** | | | |  |
| --- | --- | --- | --- | --- | --- | --- | --- | --- | --- | --- | --- | --- | --- | --- | --- | --- | --- | --- | --- | --- | --- |
| **MP_SP_SSP** | **SP_1_** | **SP_2_** | **SP_3_** | **Mean** | **SP_1_** | **SP_2_** | **SP_3_** | **Mean** | **SP_1_** | **SP_2_** | **SP_3_** | **Mean** | **SP_1_** | **SP_2_** | **SP_3_** | **Mean** | **SP_1_** | **SP_2_** | **SP_3_** | **Mean** | **Sub-Sub Plot Mean** |
| SSP_1_ | 731(139) | 774(77.49) | 810(44.82) | 771(39.55) | 824(38.52) | 836(55.94) | 866(49.40) | 842(21.63) | 881(77.80) | 999(47.82) | 1083(108) | 988(101.4) | 810(56.21) | 927(89.48) | 984(57.69) | 907(88.71) | 784(51.35) | 869(85.08) | 905(82.69) | 853(62.13) | 872(78.47) |
| SSP_2_ | 699(141) | 739(73.56) | 785(75.88) | 741(43.03) | 807(24.30) | 812(21.57) | 839(50.32) | 819(17.21) | 885(79.31) | 944(50.90) | 988(60.77) | 939(51.68) | 800(29.46) | 867(55.83) | 922(65.49) | 863(61.09) | 727(89.53) | 793(60.75) | 874(91.23) | 798(73.63) | 831(66.35) |
| Mean | 715(22.62) | 756(24.75) | 798(17.68) | 756(21.21) | 815(12.02) | 824(16.97) | 853(19.09) | 830(16.26) | 883(2.83) | 972(38.89) | 1036(67.18) | 963(34.65) | 805(7.07) | 897(42.43) | 953(43.84) | 885(31.11) | 755(40.31) | 831(53.74) | 890(21.92) | 825(38.89) | **852(69.05)** |
| MP - Main Plot, SP - Sub Plot, SSP - Sub-Sub Plot | | | | | | | | | | | | | | | | | | | | | |

MP_1_= Sole cowpea, MP_2_= Sole Baby corn MP_3_= Cowpea + Baby corn (2:1), MP_4_= Cowpea + Baby corn (3:1), MP_5_= Cowpea + Baby corn (4:1)

SP_1_=100 % RDF, SP_2_=125% RDF, SP_3_=150% RDF

SSP_1=_0.5% CaCl_2,_ SSP_2=_1% KNO_3_

**Supplementary table 16. Critical Difference (CD) values at 5% for cowpea equivalent yield**

| **Source** | **S.E. of Difference** | **t-value at 5%** | **CD at 5%** |
| --- | --- | --- | --- |
| Main plot - MP Means | 14.19 | 2.18 | 30.92 |
| Sub plot - SP Means | 13.01 | 2.04 | 26.57 |
| Sub-Sub plot - SSP Means | 8.15 | 2.01 | 16.42 |
| SP Means at the same or different levels of MP | 29.09 | 2.04 | NS |
| MP Means at the same or different levels of SP | 27.67 | 2.08 | NS |
| SSP Means at the same level of MP | 18.22 | 2.01 | NS |
| MP Means at the same or different levels of SSP | 19.17 | 2.10 | NS |
| SSP Means at the same level of SP | 14.12 | 2.01 | NS |
| SP Means at the same or different levels of SSP | 16.40 | 2.03 | NS |
| SSP Means at the same combination of MP and SP | 31.57 | 2.01 | NS |
| SP Means at the same combination of MP and SSP | 36.66 | 2.03 | NS |
| MP Means at the same combination of SP and SSP | 35.55 | 2.05 | NS |
| NS - Non Significant, MP=Main plot, SP=Sub plot, SSP=Sub-sub plot | | | |

| **Source** | **DF** | **Sum of Squares** | **Mean Square** | **F-Ratio** | **Significant** |
| --- | --- | --- | --- | --- | --- |
| Replication | 3 | 4502.0625 | 1500.6875 | 0.6440 | NS |
| C | 3 | 819302.7814 | 273100.9271 | 117.1991 | * |
| Error(a) | 9 | 20972.0833 | 2330.2315 | . |  |
| F | 2 | 153074.0194 | 76537.0097 | 24.3895 | * |
| C*F | 6 | 4088.0915 | 681.3486 | 0.2171 | NS |
| Error(b) | 24 | 75314.6042 | 3138.1085 | . |  |
| S | 1 | 27034.5938 | 27034.5938 | 28.2728 | * |
| C*S | 3 | 133.4687 | 44.4896 | 0.0465 | NS |
| F*S | 2 | 4099.2656 | 2049.6328 | 2.1435 | NS |
| C*F*S | 6 | 11238.1719 | 1873.0286 | 1.9588 | NS |
| Error(c) | 36 | 34423.3750 | 956.2049 | . |  |
| Total | 95 | 1154182.5173 | . | . |  |

**Supplementary table 17. *ANOVA* Table of cowpea - Dependent Variable is grain yield**

**Supplementary table 18. *ANOVA* Table of baby corn - Dependent Variable is raw cob yield**

| **Source** | **DF** | **Sum of Squares** | **Mean Square** | **F-Ratio** | **Significant** |
| --- | --- | --- | --- | --- | --- |
| Replication | 3 | 90060.7578 | 30020.2526 | 1.3356 | NS |
| MP | 3 | 126361141.8620 | 42120380.6207 | 1873.9276 | * |
| Error(a) | 9 | 202293.5234 | 22477.0582 | . |  |
| SP | 2 | 684197.2708 | 342098.6354 | 11.1134 | * |
| MP*SP | 6 | 63814.5208 | 10635.7535 | 0.3455 | NS |
| Error(b) | 24 | 738781.3750 | 30782.5573 | . |  |
| SSP | 1 | 166625.0026 | 166625.0026 | 5.0265 | * |
| MP*SSP | 3 | 24250.0078 | 8083.3359 | 0.2438 | NS |
| SP*SSP | 2 | 8682.5833 | 4341.2917 | 0.1310 | NS |
| MP*SP*SSP | 6 | 3492.7500 | 582.1250 | 0.0176 | NS |
| Error(c) | 36 | 1193380.0313 | 33149.4453 | . |  |
| Total | 95 | 129536719.6849 | . | . |  |

- Significant at 5% (level of significance opted by user), NS - Non Significant, C=Intercropping system, F=Fertility level, S=Stress mitigating chemicals
p-Value < 0.05 - Significant at 5%, p-Value < 0.01 - Significant at 1%

|  | **Mean sum of square** | | | | | | | | | | | | | | | | | | | | | | |
| --- | --- | --- | --- | --- | --- | --- | --- | --- | --- | --- | --- | --- | --- | --- | --- | --- | --- | --- | --- | --- | --- | --- | --- |
| **Source** | **Cowpea** | | | | | | | | | | | | **Baby corn** | | | | | | | | | | |
|  | **DF** | **CGR** | | **RGR** | | **Plant temperature** | | **RWC** | | **Chlorophyll content** | | **Seed yield** | **CGR** | | **RGR** | | **Plant temperature** | | **RWC** | | **Chlorophyll content** | | **Cob yield** |
|  |  | **25-40 DAS** | **40-55 DAS** | **25-40 DAS** | **40-55 DAS** | **25 DAS** | **50 DAS** | **25 DAS** | **50 DAS** | **25 DAS** | **50 DAS** |  | **25-40 DAS** | **40-55 DAS** | **25-40 DAS** | **40-55 DAS** | **25 DAS** | **50 DAS** | **25 DAS** | **50 DAS** | **25 DAS** | **50 DAS** |  |
| **Year** | 1 | 2.2512^*^ | 3.41867^*^ | 0.62335 | 0.137442 | 16.1008 | 26.9625 | 61.9938 | 8.4588 | 0.02876^*^ | 0.01505 | 17748.521^*^ | 2.77008 | 5.6204 | 1.1954297 | 0.00880 | 14.9187 | 4 4.4097688 | 118.912^*^ | 20.24102 | 0.026367^*^ | 0.011102^*^ | 140887.5 |
| **Replication** | 6 | 0.8295 | 0.4095 | 0.22409 | 0.18758 | 12.2482 | 19.6414^*^ | 47.1695 | 22.6506 | 0.00525 | 0.01448 | 2930.191 | 1.70066 | 4.0052 | 1.55231^*^ | 0.43598 | 8.803576 | 2 5.6163715 | 17.3435 | 39.75261 | 0.006520 | 0.007112 | 43946.7 |
| **C** | 3 | 58.4228^**^ | 82.7450^**^ | 2.98204^**^ | 1.69790474^**^ | 5.3928 | 332.7940^**^ | 34.9980 | 286.3810^**^ | 5.39442^**^ | 7.64214^**^ | 546062.6^**^ | 1319.4** | 969.31^**^ | 3.26237^**^ | 1.04839^88^ | 5.01131 | 3 03.90369^**^ | 22.2681 | 15.64868 | 4.78457^**^ | 8.036324^**^ | 84240761^**^ |
| **Year*C** | 3 | 0.1423 | 1.1867 | 0.03185 | 0.21937 | 1.1047 | 6.2099 | 2.2994 | 3.3310 | 0.00035 | 0.03762^*^ | 2214.563 | 14.051^**^ | 14.652^**^ | 0.15846 | 0.280396 | 0.01728 | 0.1687354 | 0.71144 | 0.02549 | 0.017884 | 0.377535^**^ | 11034.9 |
| **Replication*C** | 18 | 0.8043 | 0.9405 | 0.29275 | 0.27350 | 6.58598 | 7.0721 | 21.0676 | 13.9747 | 0.00670 | 0.00291 | 4344.941 | 0.9217 | 4.967** | 0.48458 | 0.3733 | 4.23254 | 9.8784086 | 26.4588 | 15.22011 | 0.004789 | 0.002988 | 32623.7 |
| **F** | 2 | 79.2875^**^ | 57.6114^**^ | 5.03011^**^ | 0.29828 | 19.7452 | 5.5848 | 21.7544 | 46.2747 | 0.17655^**^ | 0.20157^**^ | 152618.4^**^ | 166.21^**^ | 389.34^**^ | 0.28159 | 4.6639^**^ | 0.02959 | 6.3113021 | 33.0491 | 17.48974 | 0.131647^*^ | 0.201564^**^ | 684197.3^**^ |
| **Year*F** | 2 | 0.0257 | 0.9283 | 0.09829 | 0.07274 | 0.5227 | 9.8447 | 0.1440 | 0.2904 | 0.00278 | 0.00523 | 1763.474 | 0.0050 | 0.0093 | 0.05893 | 0.00814 | 0.52461 | 5.8154688 | 0.40349 | 16.49224 | 0.002002^**^ | 0.005227 | 2867.3 |
| **Replication*F** | 12 | 0.7614 | 0.3944 | 0.50831 | 0.11760 | 17.4609^**^ | 7.8461 | 27.4019 | 23.55165 | 0.00663^**^ | 0.01025 | 2499.514 | 2.1872 | 3.1345 | 1.27857^*^ | 0.20508 | 23.5957 | 6.5222049 | 23.5546 | 28.4439 | 0.004204 | 0.010961 | 30414.8 |
| **F*C** | 6 | 0.6127 | 1.9932^*^ | 0.33889 | 0.19263 | 7.5447 | 17.1580 | 2.9169 | 3.2521 | 0.00174 | 0.00164 | 1364.345 | 3.0641 | 15.95^**^ | 0.28127^*^ | 0.8261^**^ | 7.96783^*^ | 12.002968 | 18.4661 | 6.62585 | 0.001030 | 0.001641 | 21271.5 |
| **Year* F*C** | 6 | 0.0605 | 0.5138 | 0.13363 | 0.07877 | 0.9650 | 6.1794 | 0.9120 | 0.4241 | 0.00393 | 0.00114 | 3352.432 | 0.0688 | 0.0080 | 0.23025 | 0.00738 | 0.16695 | 5.5988021 | 1.08800 | 17.9089 | 0.001599 | 0.001140 | 4724.0 |
| **S** | 1 | 101.77^**^ | 42.4504^**^ | 26.4033^**^ | 0.29415 | 25.5208 | 343.5503^**^ | 110.8688 | 489.2826^**^ | 0.02453 | 0.29141^**^ | 54136.333^**^ | 128.66^**^ | 149.27^**^ | 12.5307^**^ | 1.900^**^ | 24.055^*^ | 245.9338^**^ | 86.0013 | 511.1033^**^ | 0.019000 | 0.285208^**^ | 333250.0^**^ |
| **Year*S** | 1 | 0.00044 | 1.6428 | 0.13335 | 0.14515 | 0.0133 | 0.0283 | 1.0355 | 18.4388 | 0.00220 | 0.00120 | 196.021 | 0.0720 | 0.0018 | 0.38970 | 0.00935 | 0.2760 | 1.2838021 | 10.7826 | 2.24035 | 0.002201 | 0.000833 | 24.8 |
| **Replication*S** | 6 | 0.4588380 | 0.9882 | 0.13612 | 0.22133 | 13.04098 | 17.2360 | 42.0994 | 37.1003 | 0.00474 | 0.00665 | 4078.441 | 0.2164 | 2.6532 | 0.23247 | 0.14797 | 27.948^**^ | 7.1753299 | 40.0081 | 36.9479 | 0.006421 | 0.004894 | 60060.5 |
| **C*S** | 3 | 0.0638 | 1.5484 | 0.06588 | 0.351822 | 2.9047 | 0.8088 | 12.7452 | 13.3798 | 0.00052 | 0.00253 | 88.319 | 6.9013^*^ | 5.1228 | 0.42010 | 1.3893^**^ | 1.6561 | 5.4143576 | 32.8185 | 9.52938 | 0.001322 | 0.002192 | 16166.7 |
| **F*S** | 2 | 4.3415^**^ | 2.6931^*^ | 0.76584 | 0.66078^*^ | 15.0565 | 8.3245 | 1.0252 | 14.6966 | 0.00223 | 0.00143 | 4104.036 | 2.7333 | 3.8574 | 0.26764 | 0.00040 | 18.159 | 4.2459896 | 33.1679 | 13.8544 | 0.003463 | 0.0016333 | 8682.6 |
| **S*F*C** | 6 | 0.5269 | 2.0192^*^ | 0.23257 | 0.30957 | 13.0703 | 3.1480 | 0.7599 | 12.1532 | 0.00082 | 0.00652 | 3733.648 | 1.9843 | 3.9189 | 0.66796 | 0.20161 | 11.993 | 8.0223785 | 4.78363 | 11.28218 | 0.001329 | 0.007017 | 1164.2 |
| **Year*S*C** | 3 | 0.0171 | 0.5525 | 0.02330 | 0.08623 | 0.7183 | 0.1072 | 13.1785 | 9.7305 | 0.0 0188 | 0.00197 | 27.479 | 0.0147 | 0.0016 | 0.06976 | 0.011148 | 0.9329 | 0.7421354 | 3.48866 | 1.19480 | 0.001712 | 0.001606 | 2317.6 |
| **Year S*F** | 2 | 0.0793 | 0.1013 | 0.29303 | 0.04672 | 0.1815 | 0.4109 | 2.6481 | 7.4310 | 0.00092 | 0.00248 | 3415.005 | 0.0433 | 0.0019 | 0.08529 | 0.00070 | 0.2069 | 0.3834896 | 5.35224 | 1.96773 | 0.000213 | 0.00279 | 1100.8 |
| **Year*S*F*C** | 6 | 0.0704 | 0.24130 | 0.19928 | 0.02804 | 0.6981 | 0.8714 | 0.2495 | 9.1133 | 0.00058 | 0.00630 | 813.505 | 0.0189 | 0.0017 | 0.07180 | 0.00053 | 0.3326 | 0.5709896 | 3.16939 | 1.68551 | 0.001089 | 0.00700 | 270.7 |

**Supplementary table 20a. ANOVA Table of cowpea and baby corn for different variables**

****Significant at 1% level of significance *Significant at 5% level of significance**

**Supplementary table 20b. ANOVA Table for cowpea equivalent yield**

|  | **Cowpea equivalent yield** | |
| --- | --- | --- |
| **Source** |  |  |
|  | **DF** | **Mean Square** |
| **Year** | 1 | 2464.004 |
| **Replication** | 6 | 3321.238 |
| **C** | 4 | 287790.985^**^ |
| **Year*C** | 4 | 4687.994 |
| **Replication*C** | 24 | 4225.109 |
| **F** | 2 | 247623.879^**^ |
| **Year*F** | 2 | 2185.079 |
| **Replication*F** | 12 | 2300.963 |
| **F*C** | 8 | 10460.364^**^ |
| **Year* F*C** | 8 | 2596.834 |
| **S** | 1 | 96761.504^**^ |
| **Year*S** | 1 | 185.504 |
| **Replication*S** | 6 | 5575.393 |
| **C*S** | 4 | 2023.431 |
| **F*S** | 2 | 4699.829 |
| **S*F*C** | 8 | 2747.553 |
| **Year*S*C** | 4 | 55.556 |
| **Year S*F** | 2 | 2732.129 |
| **Year*S*F*C** | 8 | 842.416 |

****Significant at 1% level of significance *Significant at 5% level of significance**
